# EZH2 enables germinal centre formation through epigenetic silencing of CDKN1A and an Rb-E2F1 feedback loop

**DOI:** 10.1038/s41467-017-01029-x

**Published:** 2017-10-12

**Authors:** Wendy Béguelin, Martín A. Rivas, María T. Calvo Fernández, Matt Teater, Alberto Purwada, David Redmond, Hao Shen, Matt F. Challman, Olivier Elemento, Ankur Singh, Ari M. Melnick

**Affiliations:** 1 Division of Hematology/Oncology, Department of Medicine, Weill Cornell Medicine, Cornell University, New York, NY 10021 USA; 2 Institute for Computational Biomedicine, Weill Cornell Medicine, Cornell University, New York, NY 10021 USA; 3 Meinig School of Biomedical Engineering, Cornell University, Ithaca, NY 14853 USA; 4 Department of Physiology and Biophysics, Weill Cornell Medicine, Cornell University, New York, NY 10021 USA; 5 Sibley School of Mechanical and Aerospace Engineering, Cornell University, Ithaca, New York, NY 14853 USA

## Abstract

The EZH2 histone methyltransferase is required for B cells to form germinal centers (GC). Here we show that EZH2 mediates GC formation through repression of cyclin-dependent kinase inhibitor *CDKN1A* (p21^Cip1^). Deletion of *Cdkn1a* rescues the GC reaction in *Ezh2* ^−/−^ mice. Using a 3D B cell follicular organoid system that mimics the GC reaction, we show that depletion of EZH2 suppresses G1 to S phase transition of GC B cells in a *Cdkn1a*-dependent manner. GC B cells of *Cdkn1a* ^−/−^ *Ezh2* ^−/−^ mice have high levels of phospho-Rb, indicating that loss of *Cdkn1a* enables progression of cell cycle. Moreover, the transcription factor E2F1 induces EZH2 during the GC reaction. *E2f1* ^−/−^ mice manifest impaired GC responses, which is rescued by restoring EZH2 expression, thus defining a positive feedback loop in which EZH2 controls GC B cell proliferation by suppressing *CDKN1A*, enabling cell cycle progression with a concomitant phosphorylation of Rb and release of E2F1.

## Abstract (web summary)

The histone methyltransferase EZH2 silences genes by generating H3K27me3 marks. Here the authors use a 3D GC organoid and show EZH2 mediates germinal centre (GC) formation through epigenetic silencing of CDKN1A and release of cell cycle checkpoints.

## Introduction

T-cell dependent antigen challenge causes a subset of mature B cells to transiently form germinal centers (GC) within lymphoid follicles^1^. B cells recruited into the GC reaction undergo clonal expansion and somatic hypermutation of their immunoglobulin loci. Those that manage to form high affinity antibodies receive signaling that drives them either to additional rounds of cell division and mutagenesis or to undergo terminal differentiation into plasma cells or memory B cells. GC B cells are unique in their ability to replicate at an accelerated rate, while undergoing somatic hypermutation^1^. Importantly, cell cycle progression is required for the enzyme AICDA to genetically modify the immunoglobulin gene variable regions^2^. Thus rapid proliferation of GC B cells is intimately linked to, and required for, the humoral immune response^1^. Despite this notion, little direct evidence exists to explain exactly how proliferation proceeds at such an exuberant pace in GC B cells. Such information would be crucial to understand the basic cellular nature of humoral immunity, as well as the biology of malignant lymphomas that arise from GC B cells and manifest their highly proliferative phenotype^3, 4^.

Silencing of cell cycle checkpoint genes is proposed to have an important function in enabling GC B cells to undergo unrestrained proliferation, although this has not been formally proven in vivo. Silencing of gene expression is mediated through epigenetic mechanisms. In the context of GC B cells, de novo epigenetic silencing of genes is mediated in large part by the Polycomb protein EZH2, which is absent from naive B cells but highly induced in the GC^5, 6^. EZH2 represses genes through its histone methyltransferase activity that catalyzes histone 3 lysine 27 trimethylation (H3K27me3), and is essential and required for B cells to form GC^7, 8^. EZH2 was reported to facilitate cell proliferation in other cell types by repressing cyclin dependent kinase inhibitors, especially *CDKN2A* (p16^Ink4a^ p14^Arf^), which is a canonical Polycomb target gene and tumor suppressor^9^. Although in vitro stimulation of *Ezh2* knockout primary B cells induced cell cycle arrest, this effect was not rescued by concomitant knockout of *Cdkn2a* ^8^. EZH2 depletion or pharmacological inhibition in GC-derived lymphoma cells also induced a profound anti-proliferative effect, and this was accompanied by more substantial derepression of *CDKN1A* (p21^Cip1^) than other cyclin-dependent kinase inhibitor genes^6, 7^. Moreover, *CDKN1A* was more potently repressed in primary GC B cells than *CDKN2A* ^6^.

Collectively these data led us to hypothesize that EZH2 is a master regulator of the striking GC proliferative phenotype, and this effect is mediated in large part through repression of *CDKN1A*. In this view, a major contribution of EZH2 to the GC reaction is to enable the characteristic proliferative phenotype of these cells, which is required for the humoral immune response. Using a combination of animal models and a newly developed organoid system that represents the phenotype and transcriptome of GC B cells, here we show that EZH2 mostly explains the GC proliferative phenotype and that this occurs in a *CDKN1A*-dependent manner, within a positive pro-proliferative regulatory feedback loop also involving E2F1 and Rb.

## Results

### Depletion of *Cdkn1a* rescues GC formation in *Ezh2*^−/−^ mice

We hypothesized that EZH2 mediated silencing of *CDKN1A* through H3K27 trimethylation might explain the proliferative GC phenotype. Previous work suggested that CDKN1A is potently repressed by EZH2 in GC-derived cells^6, 7^. We first confirmed that *CDKN1A* mRNA is expressed in purified primary human naive B cells and is differentially down-regulated in GC B cells to a greater extent that *CDKN1B*, *CDKN2A* or *CDKN2B*, whereas EZH2 expression manifests the opposite pattern (Supplementary Fig. 1A). Immunoblot analysis likewise showed virtual absence of CDKN1A in GC B cells, but robust expression in naive B cells, reciprocal to EZH2 (Supplementary Fig. 1B). Chromatin immunoprecipitation (ChIP) experiments confirmed that EZH2 binds to the *CDKN1A* promoter in primary human GC B cells and diffuse large B cell lymphoma (DLBCL) cell lines, with concordant enrichment of its H3K27me3 repressive mark (Supplementary Fig. 1C, D). Treatment of GC-derived lymphoma cell lines with EZH2 inhibitors was shown to induce *CDKN1A* mRNA expression. Here we show that EZH2 inhibitor but not an inactive control compound also consistently induced CDKN1A protein levels concordant with drug-induced depletion of EZH2 silencing mark H3K27me3 (Supplementary Fig. 1E).

**Fig. 1:**
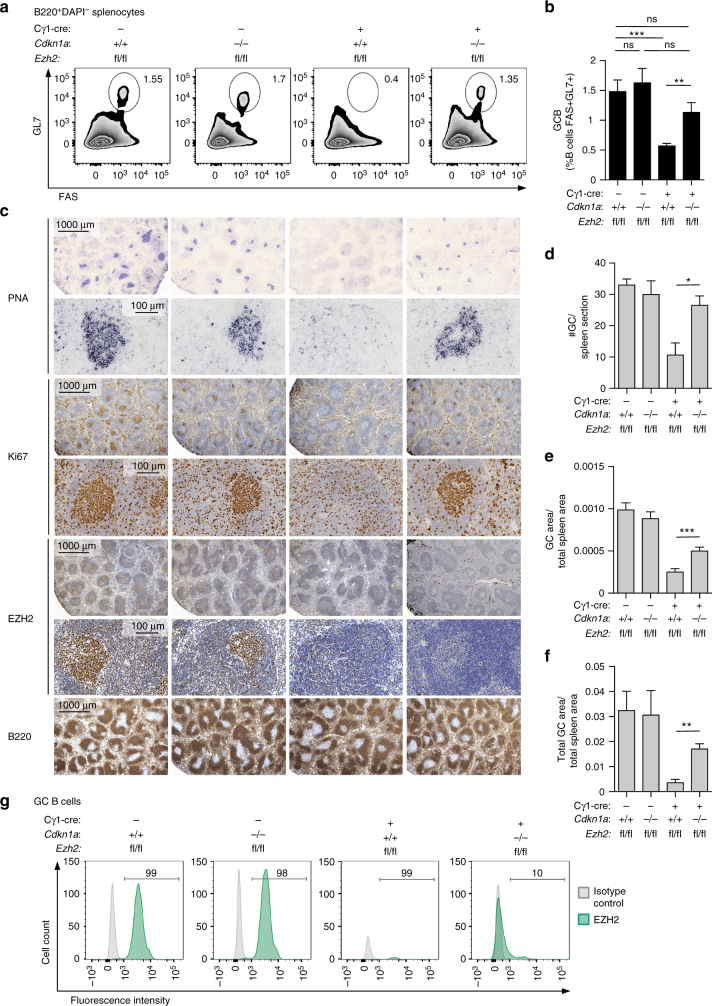
In vivo depletion of *Cdkn1a* rescues GC formation in *Ezh2* ^−/−^ mice. *Ezh2* ^*fl/fl*^, *Cdkn1a* ^−/−^, *Ezh2* ^*fl/fl*^;Cγ1-cre and *Ezh2* ^*fl/fl*^;Cγ1-cre;*Cdkn1a* ^−/−^ littermate mice were immunized with SRBC to induce germinal center (GC) formation and were killed 10 days later. **a** Flow cytometry plot of one representative mouse spleen per group. The gated area shows the percentage of GC B cells (GL7 + FAS + ) within live B cells (B220 + DAPI-, see Supplementary Fig. 2A for gating strategy). **b** Average of GC B populations of each group of mice quantified by flow cytometry as in **a** (*n* = 7 mice per group). **c** Formalin fixed paraffin embedded splenic tissue was stained for PNA, Ki67, EZH2, and B220. One representative picture of three spleens analyzed per group is shown. **d**–**f** Quantification of PNA staining from **c** (*n* = 3 spleens per group). **d** “#GC/spleen section” is the count of all GC per spleen section. **e** “GC area/total spleen area” is the quantified area of each individual GC divided by the total area of the spleen section. **f** “Total GC area/total spleen area” is the sum of all GC quantified areas in a certain section divided by the total area of that spleen section. **g** Splenocytes were permeabilized and stained for GC B and EZH2 using a fluorochrome-conjugated anti EZH2 antibody. The gated area shows the percentage of GC B cells (GL7 + FAS + ) within live B cells (B220 + DAPI-, see Supplementary Fig. 2A for gating strategy) that are EZH2 positive. The flow plot shown is representative of three spleens analyzed per group. Values in **b**, **d**, **e**, **f** are shown as mean ± SEM. *t* test, *P < 0.05, **P < 0.01, ***P < 0.001. Results are representative of a total of four independent experiments performed with different cohorts of mice. See also Supplementary Fig. 2

Next, we crossed conditional *Ezh2* ^*fl/fl*^ knockout mice^10^ with the Cγ1-cre strain, which expresses CRE recombinase in established GC B cells^11^, and these animals were crossed to *Cdkn1a* ^−/−^ mice. *Ezh2* ^*fl/fl*^;Cγ1-cre, *Cdkn1a* ^−/−^, *Ezh2* ^*fl/fl*^;Cγ1-cre;*Cdkn1a* ^−/−^ double knockout and *Ezh2* ^*fl/fl*^ control mice were immunized with the T cell-dependent antigen sheep red blood cells (SRBC) to induce GC formation. Mice were killed 10 days later, at which time the GC reaction is at its peak. As previously reported, *Ezh2* ^*fl/fl*^;Cγ1-cre mice displayed profound loss of GC B cells^7, 8^, whereas *Cdkn1a* ^−/−^ mice manifested normal abundance of such cells as measured by flow cytometry (FAS^+^ GL7^+^ B220^+^, Fig. 1a, b, and FAS^+^ CD38^−^ B220^+^, Supplementary Fig. 2A–C). Notably, the EZH2 null phenotype was almost fully rescued by concomitant *Cdkn1a* knockout. Thus *Ezh2* ^*fl/fl*^;Cγ1-cre;*Cdkn1a* ^−/−^ double knockout mice exhibited a comparable number of GC B cells as control animals. A similar GC rescue scenario was observed by immunohistochemical analysis using peanut agglutinin (PNA, a GC B cell marker), which revealed restored numbers and sizes of GC in the spleens of *Ezh2* ^*fl/fl*^;Cγ1-cre;*Cdkn1a* ^−/−^ double knockout mice versus *Ezh2* ^*fl/fl*^ controls (Fig. 1c–f). GC B cells in *Ezh2* ^*fl/fl*^;Cγ1-cre;*Cdkn1a* ^−/−^ mice stained positive for Ki67 consistent with rescue of the proliferative GC B cell compartment (Fig. 1c). Consistent with the notion that EZH2 is dispensable for GC formation in the absence of CDKN1A expression, *Ezh2* ^*fl/fl*^;Cγ1-cre;*Cdkn1a* ^−/−^ double knockout GC were EZH2 negative confirming that EZH2 was deleted from these cells (as shown by immunohistochemistry and flow cytometry in Fig. 1c, g). In contrast, the few residual GC B cells that were still present in *Ezh2* knockout mice were EZH2 positive, consistent with incomplete CRE-mediated excision of *Ezh2*. GC dark zone and light zone proportions were preserved in the double knockout mice, as measured by flow cytometry using CXCR4 and CD86 markers within the GC B cell gate (Supplementary Fig. 2D, E). These data indicate that GC architecture is not impaired. Moreover, *Ezh2* ^*fl/fl*^;Cγ1-cre;*Cdkn1a* ^−/−^ double knockout mice developed normal percentages of Th, Tfh, and GC-Tfh cells (Supplementary Fig. 2F, G). Induction of the GC B cell signature was preserved in the *Ezh2* ^*fl/fl*^; Cγ1-cre; *Cdkn1a* ^−/−^ double knockout mice, as shown by RNA-seq and GSEA (Supplementary Fig. 2H–J).

To determine if the double knockout GC were functional, we evaluated immunoglobulin affinity maturation by immunizing the mice with the specific antigen nitrophenyl-keyhole limpet hemocyanin (NP-KLH). We first confirmed that the *Ezh2* null phenotype was also rescued by *Cdkn1a* knockout in this immunization scenario (Supplementary Fig. 2K, L). We then evaluated the production of long-lived plasma cells and memory cells in mice that were boosted with NP-CGG 21 days after NP-KLH immunization. We evaluated the production of high affinity antibodies by performing ELISA with serum of mice bled 14, 21, 26, 35, and 60 days after NP-KLH immunization. *Ezh2* ^*fl/fl*^;Cγ1-cre mice showed reduction in high affinity IgG1, Ig2b, and IgA which was mostly restored in the double knockout mice (Supplementary Fig. 2M). Flow cytometry analysis of the splenocytes of these mice, which were killed 60 days after NP-KLH immunization, revealed a decrease of class switched memory B cells (non-GCB NP positive B cells) and NP positive plasma cells in *Ezh2* ^*fl/fl*^; Cγ1-cre mice which was rescued by *Cdkn1a* deletion (Supplementary Fig. 2N–P). Long-lived plasma cells reside in the bone marrow. Therefore bone marrow NP specific cell abundance was assessed by ELISPOT. *Ezh2* ^*fl/fl*^; Cγ1-cre mice manifest reduction in bone marrow IgG1^+^ NP specific cell population, whereas this loss of function was restored in *Ezh2* ^*fl/fl*^; Cγ1-cre; *Cdkn1a* ^−/−^ double knockout mice (Supplementary Fig. 2Q).

### *Cdkn1a*^−/−^ GC rescue linked to EZH2 methyltransferase function

**Fig. 2:**
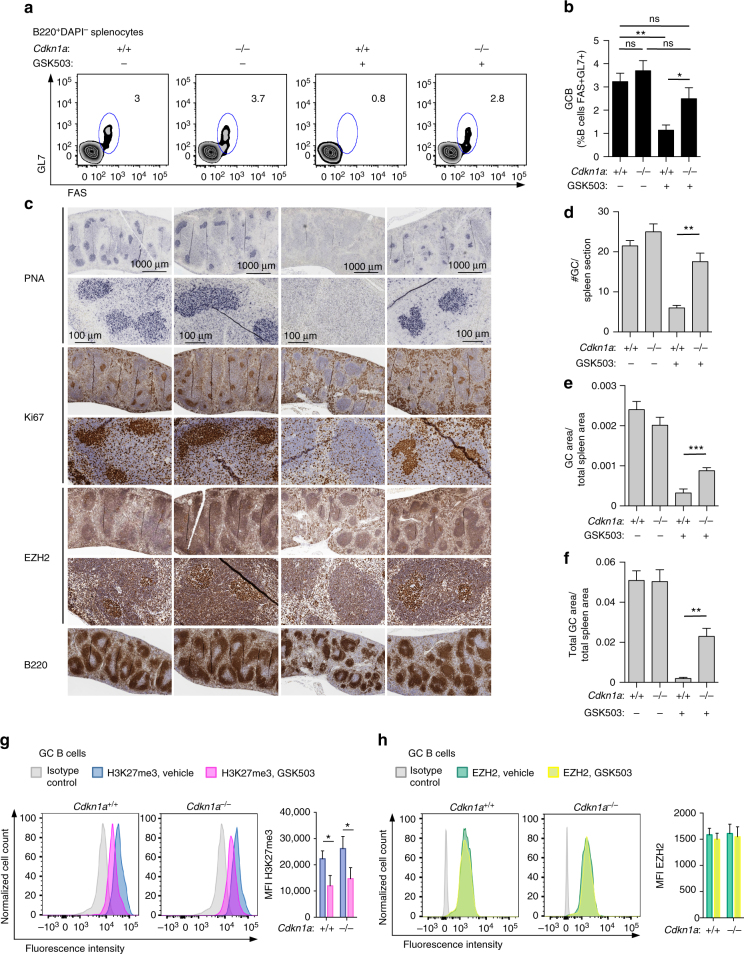
*Cdkn1a* ^−/−^ GC rescue is linked to the histone methyltransferase function of EZH2. *Cdkn1a* ^+/+^ and *Cdkn1a* ^−/−^ mice were immunized with SRBC, treated daily with GSK503 (150 mg/kg/day) or vehicle and were killed 10 days after immunization. **a** Flow cytometry plot of one representative mouse spleen per group. The gated area shows the percentage of GC B cells (GL7 + FAS + ) within live B cells (B220 + DAPI-, see Supplementary Fig. 2A for gating strategy). **b** Average of GC B populations of each group of mice quantified by flow cytometry as in **a** (*n* = 5 mice per group). **c** Formalin fixed paraffin embedded splenic tissue was stained for PNA, Ki67, EZH2, and B220. One representative picture of three spleens analyzed per group is shown. **d**–**f** Quantification of PNA staining from **c** (*n* = 3 spleens per group, see Fig. 1d–f). **g**, **h** Splenocytes from 4 WT and 4 *Cdkn1a* ^−/−^ mice were permeabilized and stained for GC B cells (GL7 + FAS + B220^+^, see Supplementary Fig. 2A for gating strategy) and H3K27me3 **g** and EZH2 **h** using fluorochrome-conjugated anti H3K27me3 and anti EZH2 antibodies, respectively. The histograms depict the mean fluorescence intensity (MFI) ± SD of H3K27me3 and EZH2 per group of mice. Values in **b**, **d**, **e**, **f** are shown as mean ± SEM. *t* test, *P < 0.05, **P < 0.01, ***P < 0.001. Results are representative of a total of three independent experiments performed with different cohorts of mice. See also Supplementary Fig. 3

To determine if the dependency of GC B cells on EZH2 for repression of CDKN1A is mediated through the enzymatic function of EZH2 we tested the effect of the specific EZH2 inhibitor GSK503 in vivo^7^. In these experiments *Cdkn1a* ^−/−^ or control mice were immunized with SRBC followed by daily treatment for 9 days with GSK503 or vehicle. Here we noted that this specific EZH2 inhibitor abrogated GC formation in WT mice but did not suppress the GC reaction in *Cdkn1a* ^−/−^ mice. This effect was manifested by normal percentage of GC B cells observed by flow cytometry (Fig. 2a, b and Supplementary Fig. 3A, B) and normal number and size of GC by immunohistochemistry (Fig. 2c–f). Importantly, we confirmed that whereas EZH2 protein abundance was not affected by the EZH2 inhibitor, H3K27me3 levels were significantly reduced as expected in both WT and *Cdkn1a* ^−/−^ treated with GSK503 (Fig. 2g, h). Therefore, the massive proliferative phenotype required for GC formation and the humoral immune response is dependent on EZH2 repression of *CDKN1A* through its enzymatic activity.

### Characterization of a B cell organoid to model the GC reaction

**Fig. 3:**
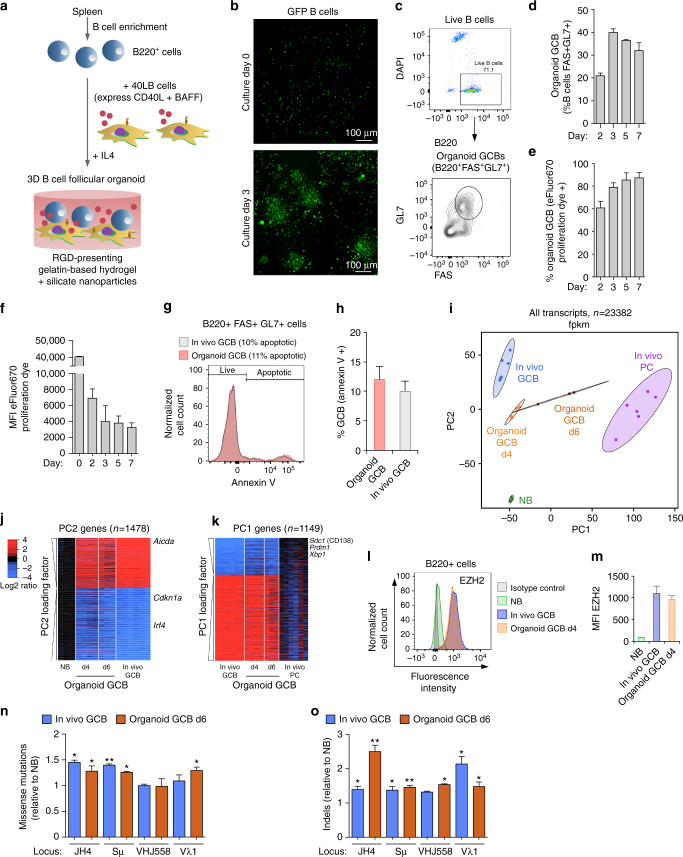
Characterization of a 3D B cell follicular organoid to model the GC reaction. **a** Scheme of 3D B cell follicular organoid fabrication. Splenic B cells are co-encapsulated with 40LB stromal cells and IL4 into an RGD-presenting nanocomposite hydrogel that contain gelatin ionically cross-linked with synthetic silicate nanoparticles. **b** Representative fluorescence pictures of splenic GFP B cells in 3D organoid culture. **c** Flow cytometry plot of a 3D B cell follicular organoid. The gated area on the top shows the live B cells (B220 + DAPI−) and on the bottom plot, the organoid GC B cells (GL7 + FAS + ) within live B cells. **d** Average of GC B populations of organoids quantified as in **c**. **e** Average of percentage of proliferating organoid GC B populations, quantified as indicated in Supplementary Fig. 4B. **f** MFI of proliferation dye from **e**. **g** Flow cytometry plot of GC B cells stained with annexinV. **h** Average of percentage of apoptotic GC B cells quantified as in **g**. **i** RNA-seq profiles of organoid GC B cells after 4 and 6 days in culture (organoid GCB d4, *n* = 4 spleens, and d6, *n* = 3 spleens) were projected into the principal component space defined by GC B cells sorted from immunized mice (in vivo GCB, *n* = 6 mice), CD138 + plasma cells (in vivo PC, *n* = 6 mice) and FAS-GL7-IgD + B220 + naive B cells (NB, *n* = 3 mice). **j**, **k** Heat maps of gene expression level of GC B cells showed in **i**, represented as log2 ratio relative to mean naive B cells **j** and to mean plasma cells **k**. PC1/2 = principal component 1/2. **l** Splenocytes were permeabilized and stained for EZH2, and co-stained for GL7, FAS, IgD and B220 to identify GC B cells (FAS + GL7 + B220 + ) and naive B cells (FAS-GL7-IgD + B220 + ). **m** MFI of EZH2 (*n* = 3 per group) from **l**. **n**, **o** The indicated immunoglobulin variable loci were sequenced from organoid or in vivo GC B and naive B cells (*n* = 3 biological replicates per group). *t* test vs. naive B cells, *P < 0.05, **P < 0.01. Values in **d**–**f**, **h**, **m**–**o** are mean ± SEM. **d**–**h**, *n* = 3 organoids per time-point. Results shown in **b**–**h** are representative of 4 independent experiments. See also Supplementary Fig. 4

To further understand the role of EZH2 as a driver of the cell cycle we next wished to explore its relation to the G1 checkpoint regulated by CDKN1A. To date, mechanistic studies in primary GC B cells have been limited by the fact that these cells are not readily amenable to being experimented with ex vivo. Moreover it is well accepted that simply activating resting B cells ex vivo does not recapitulate the GC phenotype^1^. To overcome this challenge we optimized a three-dimensional (3D) B cell follicular organoid culture system based on work published by Purwada et al.^12, 13^ and determined whether this system could mimic the phenotypic and transcriptional regulatory characteristics of primary GC B cells. This culture system provides the opportunity to generate hundreds of 3D organoids using B cells from one spleen. To provide T cell-mediated CD40 and FDC-mediated BAFF signaling, we used BALB/c 3T3 fibroblasts stably transfected with both CD40L and BAFF (40LB cells^14^). Primary B cells were isolated from unstimulated mouse spleens and co-encapsulated with 40LB stromal cells into an Arg-Gly-Asp (RGD)-presenting hydrogel organoid that mimics the extracellular matrix network within lymphoid follicles where GC form. These hydrogels contain gelatin ionically cross-linked with synthetic silicate nanoparticles, which remain stable in culture and do not liquefy at 37 °C (Fig. 3a). Recombinant murine IL4 was added to the media where the hydrogel capsules were embedded. These materials are engineered to resemble the microarchitecture of lymphoid tissue, which provides structural stability while allowing cell proliferation. Primary B cells encapsulated in these cultures formed large cell clusters within 3 days (Fig. 3b). Approximately 40% of these manifested the characteristic GC B cell immunophenotype (FAS^+^ GL7^+^ B220^+^, and reduced CD38 staining Fig. 3c, d and Supplementary Fig. 4A). Consistent with the GC phenotype the FAS^+^ GL7^+^ B220^+^ cells also exhibited massive proliferation, as measured by eFluor 670 dye dilution (Fig. 3e, f and Supplementary Fig. 4B). GC B cells typically undergo apoptosis ex vivo, however in this 3D organoid context the rate of apoptosis was similar to that of GC B cells in vivo ( ~ 10% of cells annexin V positive, Fig. 3g, h).

To determine if 3D organoid GC B cells manifested the characteristic GC B cell transcriptome, we compared and contrasted RNA-seq profiles from FAS^+^ GL7^+^ B220^+^ organoid GC B cells at days 4 and 6, vs. GC B cells sorted from immunized mice. RNA-seq was also performed in the same purified naive B cells used to seed the organoids, as well as plasma cells (CD138^+^) sorted from immunized mice. RNA-seq profiles of organoid GC B cell were projected into the principal component space defined by in vivo GC B cells, plasma cells and naive B cells. Notably, we found that organoid GC B cells at day 4 of culture shift toward the in vivo GC B cell profile, indicating that these cells have acquired the GC transcriptional program (Fig. 3i). Late-stage organoid GC B cells (day 6) shift towards the plasma cell gene expression profile (Fig. 3i). A similar pattern was observed using gene ontogeny tree analysis as an alternative approach (Supplementary Fig. 4C). Both results suggest that early-stage organoid B cells are closer in identity to GC B cells in vivo, whereas late-stage organoid GC B cells have begun to adopt the hallmark gene expression pattern associated with terminal differentiation. Analysis of the genes that drive the expression differences captured by our principal component analysis, showed that organoid GC B cells feature a gene expression pattern similar to in vivo GC B cells, as opposed to naive B cells and plasma cells (Fig. 3j, k). Using an additional orthogonal approach we observed that the in vivo GC B cell signature is highly enriched in the gene expression profiles of organoid day 4 GC B cells as compared with naive B cells (GSEA FDR q < 0.001; Supplementary Fig. 4D). GC hallmark genes, such as *Ezh2*, *Bcl6* and *Aicda* were highly induced in organoid GC B cells after 4 days, measured by qPCR, while *Cdkn1a* was downregulated as compared with naive B cells (Supplementary Fig. 4E). We found that EZH2 and BCL6 proteins are also induced in GC organoids, at levels comparable to in vivo GC B cells by flow cytometry (Fig. 3l, m and Supplementary Fig. 4F). Notably, in the absence of the hydrogel nanoparticle matrix GC B cells (grown in “2D” conditions) do not proliferate as efficiently, are more apoptotic and manifest transcriptional profiles more distant to in vivo GC B cells than their 3D counterparts, highlighting the importance of using the full system to achieve this phenotype (Supplementary Fig. 4G–J).

Whereas B cells in culture readily undergo class switch recombination, the hallmark of GC B cells is somatic hypermutation. To further assess the extent to which GC organoids mimic GC biology, we evaluated whether the immunoglobulin gene variable regions manifest evidence of somatic hypermutation. We amplified immunoglobulin variable loci by PCR from purified naive B cells (culture day 0), GCBs sorted from ex vivo cultures for 6 days, and naive B cells and GCBs sorted from immunized mice. Analysis of sequencing data revealed a significant increase in indels and missense mutations in organoid GC B cells as compared with naive B cells, similar to in vivo GC B cells^15^ (Fig. 3n, o). Taken together, these features indicate that our GC organoid system reproduces core aspects of the GC B cell phenotype and hence is a suitable model to study GC B cell functions of EZH2.

### *CDKN1A* repression is required for GC B cell cycle progression

**Fig. 4:**
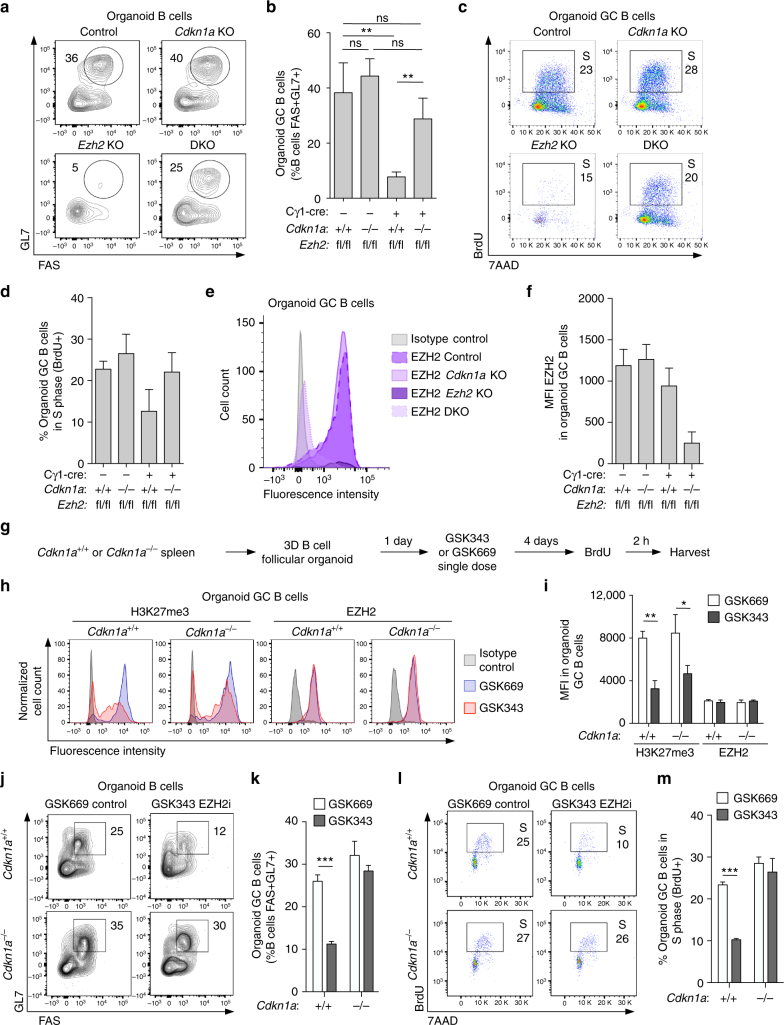
*CDKN1A* repression by EZH2 is required for GC B cell cycle progression. **a**–**f** Organoids were generated using B cells isolated from *Ezh2* ^*fl/fl*^;Cγ1-cre, *Cdkn1a* ^−/−^, *Ezh2* ^*fl/fl*^;Cγ1-cre;*Cdkn1a* ^−/−^ and *Ezh2* ^*fl/fl*^ control mice (*n* = 3 mice per group) and were harvested for flow cytometry analysis after 4 days in culture. **a** Flow cytometry plots of representative organoids from each genotype. The gated area shows the percentage of organoid GC B cells (GL7 + FAS + ) within live B cells (B220 + DAPI−). **b** Average of percentage of organoid GC B populations quantified by flow cytometry as in **a** (*n* = 3 biological replicates per group). **c** Organoids received a BrdU pulse of 2 h before harvest. Cell cycle was analyzed by BrdU staining and 7AAD to measure DNA content. The representative gated area shows the percentage of organoid GC B cells (GL7 + FAS + B220 + DAPI−) that are in S phase (BrdU + ). **d** Average of percentage of organoid GC B populations in S phase of each group of genotype quantified by flow cytometry as in **c** (*n* = 3 biological replicates per group). **e** Organoid cells were permeabilized and stained for EZH2 and GC markers GL7, FAS, and B220 to identify organoid GC B cells. The flow cytometry plot shows one representative sample per genotype group. **f** MFI of EZH2 in organoid GC B cells (*n* = 3) quantified by flow cytometry as in **e**. **g**–**m** Organoids were generated using B cells isolated from 3 *Cdkn1a* ^+/+^ and 3 *Cdkn1a* ^−/−^ mice. **g** Scheme of EZH2 inhibitor GSK343 and BrdU treatment ex vivo. **h** Organoid cells were permeabilized and stained for EZH2, H3K27me3 and GC markers GL7, FAS, and B220. The flow cytometry plot shows one representative sample per group. **i** MFI of H3K27me3 and EZH2 in organoid GC B cells quantified by flow cytometry as in **h** (*n* = 3 biological replicates per group). **j**–**m** Analysis of percentages of organoid GC B cells and percentages of these cells in S phase was performed, as described in **a**–**d** (*n* = 3 biological replicates per group). **k**, **m** show mean ± SEM. Values in **b**, **d**, **f**, **i** are mean ± SD. *t* test, *P < 0.05, **P < 0.01, ***P < 0.001. Results are representative of a total of three independent experiments

We next wished to validate whether the 3D organoid GC B cells could recapitulate the phenotype observed in *Ezh2* ^*fl/fl*^;Cγ1-cre;*Cdkn1a* ^−/−^ double knockout mice in vivo. We therefore generated organoids from naive B cells isolated from *Ezh2* ^*fl/fl*^;Cγ1-cre, *Cdkn1a* ^−/−^, *Ezh2* ^*fl/fl*^;Cγ1-cre;*Cdkn1a* ^−/−^ double knockout and *Ezh2* ^*fl/fl*^ control mice. Strikingly, the organoid system recapitulated the significant GC B cell loss phenotype induced by conditional deletion of *Ezh2* in vivo (Fig. 4a, b). *Cdkn1a* ^−/−^ alone had no impact on organoid GC formation. However the *Ezh2* null phenotype was largely rescued when organoids were generated from *Ezh2* ^*fl/fl*^;Cγ1-cre;*Cdkn1a* ^−/−^ double knockout B cells (Fig. 4a, b). Regulation of CDKN1A is expected to affect the G1 to S phase transition of GC B cells. Accordingly, we observed a reduction in the proportion of GC B cells entering S phase in *Ezh2* ^*fl/fl*^;Cγ1-cre organoids. In contrast, cell cycle progression was rescued in *Ezh2* ^*fl/fl*^;Cγ1-cre;*Cdkn1a* ^−/−^ double knockout organoids (Fig. 4c, d). We noted that reduction of S phase GC B cells in *Ezh2* ^*fl/fl*^;Cγ1-cre organoids did not reach statistical significance (Fig. 4d), perhaps because of incomplete CRE-mediated excision of *Ezh2* in the residual GC B cells. Indeed, similar to what we observed in mice (Fig. 1g), the residual organoid GC B cells were also EZH2 positive. However, flow cytometry analysis confirmed EZH2 expression in *Ezh2* ^*fl/fl*^ control and *Cdkn1a* ^−/−^ GC B cells, and its absence in *Ezh2* ^*fl/fl*^;Cγ1-cre; *Cdkn1a* ^−/−^ organoid GC B cells (Fig. 4e, f).

To better control the timing of EZH2 loss of function and the extent to which EZH2 is inactivated among the population of GC B cells, we exposed organoids to the specific EZH2 inhibitor GSK343. After testing different schemes of EZH2 inhibitor treatment ex vivo, we selected an optimal schedule that would allow B cells to differentiate into GC B cells before exposure to EZH2 inhibition and proliferation arrest. Splenic B cells from WT and *Cdkn1a* ^−/−^ mice (*n* = 3 each) were encapsulated into 3D organoids, treated next day with 2 µM GSK343 or control compound GSK669 and incubated during 4 days in culture, followed by a 2 h BrdU pulse before harvest (Fig. 4g). GSK343 significantly inhibited EZH2 methyltransferase activity in organoid GC B cells, as measured by H3K27me3 flow cytometry, without affecting EZH2 levels (Fig. 4h, i). Similar to its effect in vivo, GSK343 caused a significant reduction in the percentage of GC B cells in organoids (*t* test, *P* < 0.001) as determined by flow cytometry (Fig. 4j, k). In marked contrast, EZH2 inhibitors did not cause a reduction in GC B cells, when organoids were formed from B cells obtained from *Cdkn1a* ^−/−^ mice. Analysis of cell cycle progression based on BrdU incorporation revealed that the EZH2 inhibitor strongly and significantly suppressed G1 to S phase transition of WT GC B cells (*t* test, *P* < 0.001). However there was no such reduction in G1-S transition in the GC organoids generated from *Cdkn1a* ^−/−^ B cells (Fig. 4l, m). Collectively these data indicate that EZH2 is required for GC proliferation through its repression of *CDKN1A*, which allows unrestricted G1-S transition in these cells. These results also point to the suitability of our GC organoids for enabling biologically relevant mechanistic studies in primary GC B cells ex vivo.

### EZH2 is required to enable Rb phosphorylation in GC B cells

**Fig. 5:**
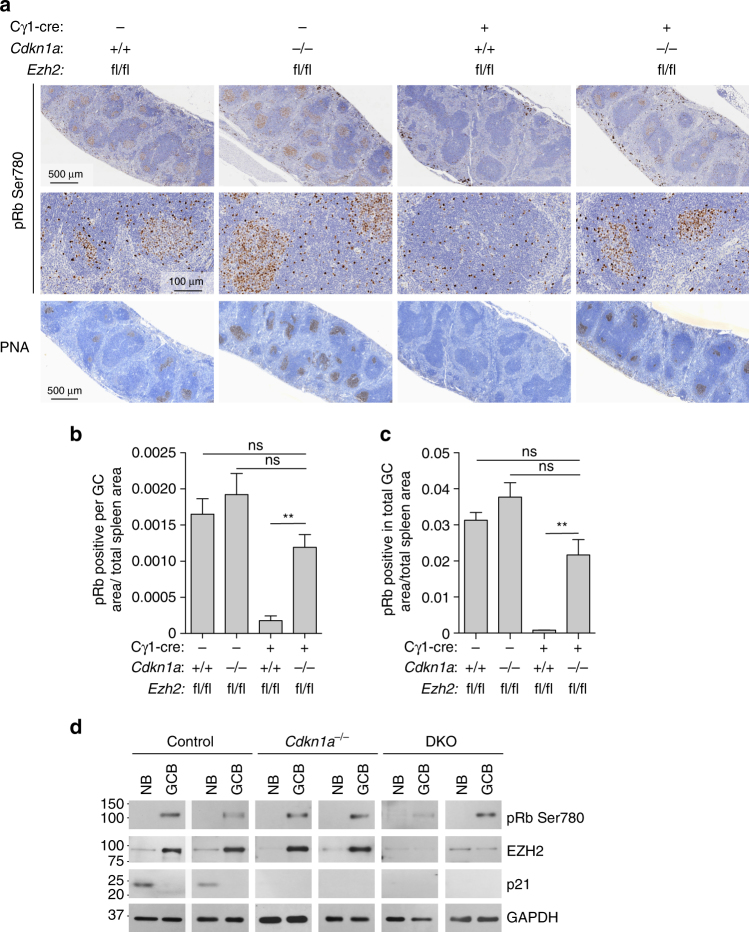
EZH2 is required to enable Rb phosphorylation in GC B cells. **a** Formalin fixed paraffin embedded splenic tissue from mice shown in Fig. 1 was stained for phospho Rb Ser780 and PNA. One representative picture of three spleens analyzed per genotype group is shown. **b**, **c** Quantification of phospho Rb Ser780 staining from **a** (*n* = 3 spleens per group). **b** “pRb positive per GC area/total spleen area” is the quantified area of each individual GC divided by the total area of the spleen section. **c** “pRb positive in total GC area/total spleen area” is the sum of all GC quantified areas in a certain section divided by the total area of that spleen section. Values are shown as mean of triplicates ± SEM. *t* test, **P < 0.01. **d** Immunoblotting of whole cell lysates from naive B cells (FAS^−^ GL7^−^ IgD^+^ B220^+^) and GC B cells (FAS^+^ GL7^+^ B220^+^) sorted from 2 *Cdkn1a* ^−/−^, 2 *Ezh2* ^*fl/fl*^;Cγ1-cre;*Cdkn1a* ^−/−^ double knockout (DKO) and 2 *Ezh2* ^*fl/fl*^ control mice immunized with SRBC for 10 days. GAPDH was used as loading control. The faint EZH2 band in DKO GC B cells is due to the few residual GC B cells that were still EZH2 positive, consistent with incomplete CRE-mediated excision of *Ezh2*. Numbers on the left indicate molecular weight in kDa. See Supplementary Fig. 6A for uncropped scans of the western blots

CDKN1A inhibits the activity of CDK4/6 and CDK2, the kinases responsible for the phosphorylation of the retinoblastoma (Rb) protein. Specifically, phosphorylation of Rb on Ser780 allows dissociation of the transcription factor E2F from the Rb/E2F complexes^16, 17^ and the subsequent transcription of E2F target genes, which are responsible for the progression through the G1 phase and S transition^18, 19^. To further explore the impact of EZH2 on these cell cycle events related to CDKN1A function, we next evaluated the phosphorylation of Rb on Ser780 by immunohistochemistry in the spleen of *Ezh2* ^*fl/fl*^;Cγ1-cre, *Cdkn1a* ^−/−^, *Ezh2* ^*fl/fl*^;Cγ1-cre;*Cdkn1a* ^−/−^ double knockout and *Ezh2* ^*fl/fl*^ control mice immunized with SRBC after 10 days. We noted high density of phospho Rb positive cells inside GC, corresponding to the highly proliferative GC B cells (Fig. 5a–c), and similar to Ki67 staining pattern in spleens (see Fig. 1c). In contrast *Ezh2* ^*fl/fl*^;Cγ1-cre knockout spleens lacked the dense population of phospho Rb positive cells within follicles consistent with loss of GC B cells in these animals. However the GC Rb phosphorylation pattern was restored in GC B cells from *Ezh2* ^*fl/fl*^;Cγ1-cre;*Cdkn1a* ^−/−^ double knockout mice, approaching similar levels to those observed in *Ezh2* ^*fl/fl*^ or *Cdkn1a* ^−/−^ control mice (Fig. 5a–c). We confirmed these results by performing western blot of pRb (Ser780) in naive B (FAS^−^ GL7^−^ IgD^+^ B220^+^) and GC B cells (FAS^+^ GL7^+^ B220^+^) sorted from *Cdkn1a* ^−/−^, *Ezh2* ^*fl/fl*^; Cγ1-cre; *Cdkn1a* ^−/−^ double knockout and *Ezh2* ^*fl/fl*^ control mice immunized with SRBC for 10 days (Fig. 5d).

### E2F1 induces the expression of EZH2 in GC B cells

**Fig. 6:**
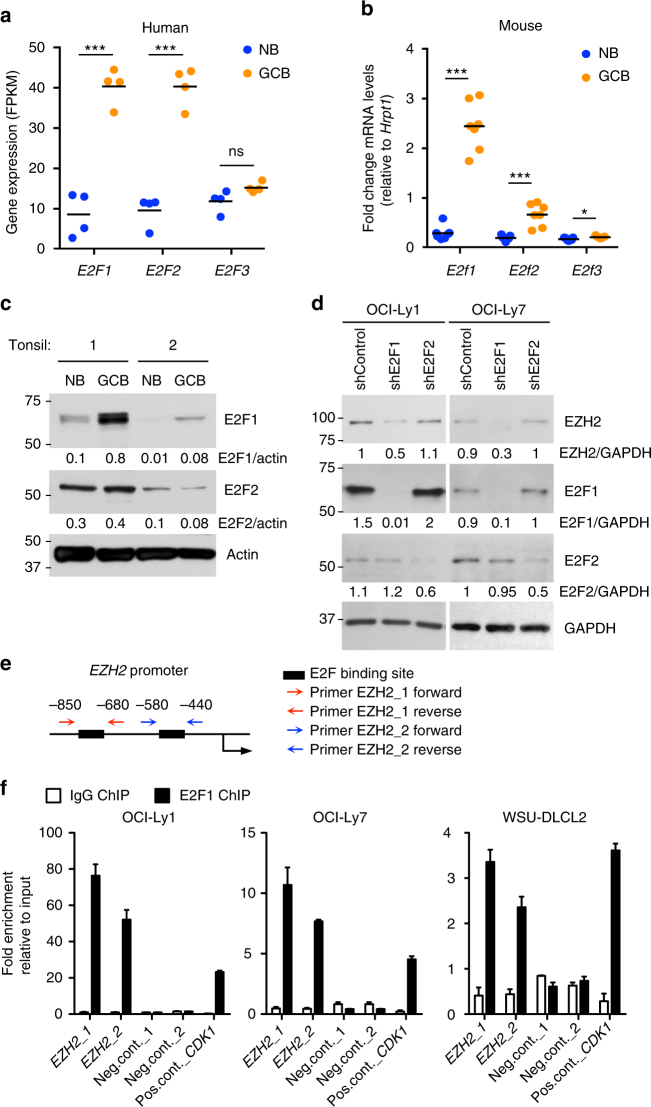
E2F1 induces the expression of EZH2 in GC B cells. **a** Expression level in FPKM of *E2F* genes in naive B and GC B cells from 4 human tonsils. Horizontal black lines represent mean. *t* test, ***P < 0.001. **b** RT-qPCR of *E2f* in GCBs (FAS + GL7 + B220 + ) and naive B cells (FAS-GL7-IgD + B220 + ) sorted from 7 WT mice. Horizontal black lines represent mean fold change mRNA levels normalized to *Hprt1*. *t* test, ***P < 0.001. **c** Immunoblotting of whole cell lysates from naive B and GC B cells from two human tonsil samples. Actin was used as loading control. The western blot shown is representative of a total of five tonsils analyzed. See Supplementary Fig. 6B for uncropped scans of the western blots. **d** Representative immunoblotting of whole cell lysates from OCI-Ly1 and OCI-Ly7 cells expressing shRNAs against E2F1, E2F2 or control. GAPDH was used as loading control. The experiment was repeated two more times with similar results. See Supplementary Fig. 6C for uncropped scans of the western blots. **e** Schematic representation of the genomic region corresponding to the promoter of *EZH2*. The black boxes represent a region of putative E2F binding sites. Two pairs of primers were design to flank the putative E2F binding sites. **f** E2F1 and control IgG qChIP was performed in the indicated cell lines using the primers described in **e**. As negative control qPCR was performed using primers for two regions in chromosomes 6 and 7 where no enrichment of transcription factors was found by ChIP-seq read density in OCI-Ly1 and OCI-Ly7 cells. As positive control qPCR was performed using primers flanking an E2F binding site at the promoter of *CDK1*. Fold enrichment was normalized to the input. Values are shown as mean of technical triplicates ± SEM. The experiment shown is representative of three independent ChIPs performed with OCI-Ly1 and OCI-Ly7, and two with WSU-DLCL2

Phosphorylation of Rb facilitates the activity of E2F transcription factors. EZH2 was previously shown to be a direct target of E2F factors^20^. E2F1, E2F2 and, to a lesser extent, E2F3 bind to E2F-binding sites located in the promoter of EZH2 to activate its transcription^20^. To evaluate if E2F transcription factors also regulate EZH2 expression in GC B cells, we first analyzed their expression in purified primary B cells from human tonsils and murine spleens. We found that *E2F1* and *E2F2* are highly expressed and up-regulated in GC B cells as compared with naive B cells (Fig. 6a, b). However, immunoblot analysis showed that at the protein level only E2F1 is strongly induced in human tonsilar GC B cells (Fig. 6c). While shRNA-mediated silencing of E2F1 in GC-derived DLBCL cell lines caused a reduction in EZH2 protein expression, it was not affected by E2F2 knockdown (Fig. 6d). We next evaluated E2F1 recruitment to E2F-binding sites located within the *EZH2* promoter by qChIP in three GC-derived DLBCL cell lines (Fig. 6e). As a positive control we tested E2F1 ChIP enrichment at the *CDK1* promoter, a known target of E2F1. We found that E2F1 was strongly enriched at the *EZH2* promoter in these GC-derived B cells, to similar or even higher levels than *CDK1* promoter (Fig. 6f).

### E2F1 is required for GC formation in an EZH2 dependent manner

**Fig. 7:**
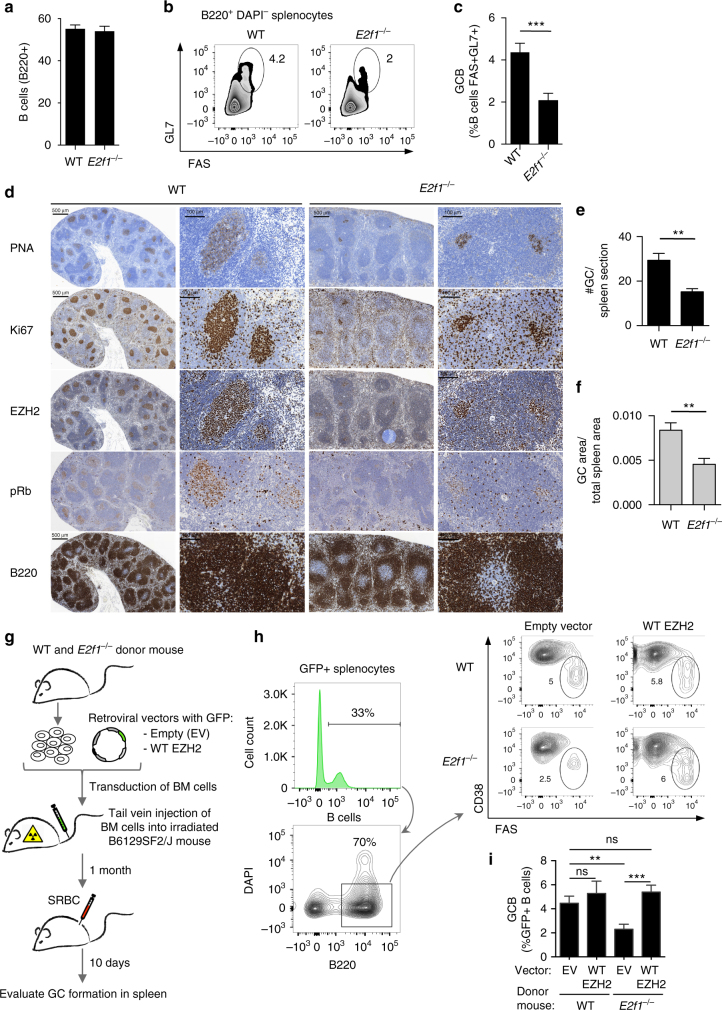
E2F1 is required for GC formation in an EZH2 dependent manner. **a**–**f** *E2f1* ^−/−^ and control WT mice were immunized with SRBC to induce GC formation and were killed 10 days later. **a** Average of percentage of B cells (B220 + ) within live splenocytes (DAPI−) of each group of mice (*n* = 9 per group). **b** Flow cytometry plot of one representative mouse spleen per group. The gated area shows the percentage of GC B cells (GL7 + FAS + ) within live B cells (B220 + DAPI−, see Supplementary Fig. 2A for gating strategy). **c** Average of percentage of GC B populations of each group quantified by flow cytometry as in **b** (*n* = 9 per group). **d** Formalin fixed paraffin embedded splenic tissue was stained for PNA, Ki67, EZH2, phospho Rb Ser780 and B220. One representative picture of 5 spleens analyzed per group is shown. **e**, **f** Quantification of PNA staining from **d** (*n* = 5 spleens per group). **e** “#GC/spleen section” is the count of all GC per spleen section. **f** “GC area/total spleen area” is the quantified area of each individual GC divided by the total area of the spleen section. Results shown in **a**–**f** are representative of a total of three independent experiments performed with different cohorts of mice. **g** Bone marrow transplantation was performed using *E2f1* ^−/−^ and WT donor mice. Each recipient group consisted of 8 mice. BM, bone marrow. **h** Gating strategy used to analyze the GFP positive GC B cells from transplanted mice. Representative flow cytometry plots showing the gating on GC B cells (GL7 + FAS + ) within live GFP B cells (B220 + DAPI-GFP + ) per mouse group. **i** Average of percentage of GFP positive GC B cells in each transplant group (*n* = 8) quantified by flow cytometry as shown in **h**. Values in **a**, **c**, **e**, **f**, **i** are shown as mean ± SEM. *t* test, **P < 0.01, ***P < 0.001. Results shown in **a**–**f** are representative of a total of four independent experiments

If E2F1 helps to drive EZH2 expression in GC B cells, it might be expected that E2F1 loss of function would impair GC formation. To address this question we compared and contrasted GC formation in the spleens of *E2f1* deficient and WT control mice immunized with SRBC and were killed 10 days later. *E2f1* deletion had no impact on total numbers of splenic B cells (Fig. 7a). In contrast GC formation was impaired in *E2f1* ^−/−^ mice as manifested by significantly reduced numbers of GC B cells measured by flow cytometry (*t* test, *P* < 0.001, Fig. 7b, c). Immunohistochemical analysis further revealed reduced number and size of GC in *E2f1* ^−/−^ mice versus controls (*t* test, *P* < 0.01, Fig. 7d–f). Residual GC in *E2f1* ^−/−^ mice stained positive for EZH2, Ki67 and phospho Rb (Fig. 7d), suggesting that *Ezh2* expression is partially compensated for by other transcription factors.

To determine if this impaired GC phenotype was due to a lack of induction of *Ezh2* by E2F1, we restored EZH2 expression in *E2f1* ^−/−^ mice by exogenously expressing EZH2. For this purpose we transduced bone marrow of *E2f1* ^−/−^ or WT control donor mice with retrovirus encoding EZH2-GFP or GFP alone expressing retrovirus, transplanted them into lethally irradiated recipients and once engrafted, assessed the GC reaction ten days after immunization with SRBC (Fig. 7g). Analysis of GFP positive GC B cells by flow cytometry (FAS^+^ CD38^−^ B220^+^ GFP^+^) showed a reduced number of GC B cells in mice transplanted with *E2f1* ^−/−^ + GFP alone as compared with mice that received WT bone marrow + GFP virus (*t* test, *P* < 0.01, Fig. 7h, i). In contrast, EZH2 expression in *E2f1* ^−/−^ mice completely restored the number of GC B cells (*E2f1* ^−/−^ + GFP alone vs. *E2f1* ^−/−^ + EZH2, *t* test, *P* < 0.001, Fig. 7h, i). Taken together these data indicate that proliferation of GC B cells is dependent on an E2F1-EZH2-CDKN1A-Rb-E2F1 positive feedback loop (Supplementary Fig. 5).

## Discussion

Herein we provide direct evidence that an EZH2 dependent positive feedback loop drives the characteristic proliferative phenotype of GC B cells. This is important not only to understand the cell biology of the GC reaction but also because proliferation is intimately linked and required for immunoglobulin affinity maturation^1, 2^. Our data delineate a model whereby EZH2 drives unrestricted cycling of GC B cells by directly repressing cyclin-dependent kinase inhibitor gene *CDKN1A*. This leads to Rb phosphorylation, which in turn enables the transcription factor E2F1 to drive cell cycle forward, as well as to further induce EZH2 expression, hence establishing a positive feedback loop that enables clonal expansion and thus immunoglobulin affinity maturation during the humoral immune response (Supplementary Fig. 5). Some of the striking features of this feedback mechanism include the rescue of the EZH2 GC null phenotype by concomitant *Cdkn1a* knockout, the rescue of impaired GC response due to *E2f1* knockout by adding back EZH2, and the requirement for EZH2 repression of *CDKN1A* to drive G1-S transition in GC B cells. Other E2F family proteins might also contribute to some extent, as suggested by the residual GC observed in *E2f1* knockout mice.

These events play out in established GC B cells, after the initial stage of B cell activation, where MYC is transiently expressed and likely prepares B cells for the GC proliferative burst^21, 22^. Importantly MYC can induce expression and activation of E2F1 by releasing it from Rb and enhancing its activity through CDK-dependent mechanisms^23–25^. These actions likely set the stage for the E2F1-EZH2-CDKN1A-Rb positive feedback loop. Notably, MYC is silenced in (EZH2-dependent) proliferating centroblasts, due to the actions of BCL6 transcriptional repressor^21, 26^. The compartmentalization of MYC and EZH2 may avoid the potential oncogenic hazard of enabling (MYC driven) cell growth pathways to function in cells with unrestrained proliferative potential. Collectively, the data suggest a scenario whereby activated B cells are not fully licensed to undergo the proliferative burst until EZH2 “pulls the brake” from these by silencing *CDKN1A* so that they can now replicate without cell cycle checkpoint “friction”.

It is important to note that EZH2 represses other cyclin-dependent kinase inhibitors including *CDKN2A* and *CDKN1B* in GC B cells. Yet *CDKN1A* was the most upregulated among these genes upon EZH2 knockdown^6, 7^. Among these cyclin-dependent kinase inhibitors, CDKN1A is also the one that undergoes the most profound change in levels during the transition from naive to GC B cell (as shown in Supplementary Fig. 1A). As noted previously, *CDKN2A* is the canonical Polycomb repressed cell cycle checkpoint gene. Although Caganova et al.^8^ showed that *Cdkn2a* deletion was unable to rescue the proliferative phenotype in *Ezh2* knockout B cells ex vivo, this does not preclude that it could still contribute to cell cycle in vivo. As compared with other cyclin-dependent kinase inhibitor genes, *CDKN1A* is a more general inhibitor that targets multiple CDKs^27^, which may explain why it is that repression of this particular target so potently rescues the EZH2 null phenotype in GC formation. Nonetheless, the data do not rule out a potential although smaller contribution of *CDKN2A* and *CDKN1B* to the actions of EZH2, which perhaps could explain why GC rescue was not 100% with *CDKN1A* knockout. It is worth noting that the BCL6 transcriptional repressor can also repress *CDKN1A* ^28, 29^ in cooperation with EZH2^30^. Yet in contrast to EZH2, *CDKN1A* knockout did not rescue loss of GC in mice downstream of BCL6 loss of function (data not shown), suggesting that BCL6 repression of other genes is more critical to its actions in GC formation.

To date, a major impediment to detailed mechanistic studies in GC B cells is the lack of experimental systems that sufficiently recapitulate the biology of this complex biological system^1^. Although it is possible to activate B cell ex vivo, the resulting proliferating and class switching B cells are more akin to the non-GC extrafollicular response^1^. Yet to study the mechanism of action of GC factors such as EZH2 in living, healthy and replicating GC B cells requires the ability to manipulate and generate such cells in a tractable system. Here we generated, characterized and refined an ex vivo 3D organoid system that more faithfully recapitulates key features of GC B cells. These 3D organoid GC B cells manifest the characteristic immunophenotype and transcriptional signature of GC B cells in vivo. Importantly, GC B cells isolated from murine lymphoid tissues cease proliferating and undergo apoptosis ex vivo. However virtually 100% of the organoid GC B cells were proliferating and their death rate was no different than GC B cells in vivo. The organoid B cells also upregulate AICDA, EZH2, and BCL6 and manifest immunoglobulin gene somatic hypermutation. Most strikingly and of great significance for use of this model for mechanistic studies, the organoid recapitulates GC specific activation of a conditional allele driven by Cγ1-cre (*Ezh2*), as well as its downstream GC-specific phenotypic consequences, and its rescue by a second genetic manipulation (in this case *Cdkn1a* knockout). Along these lines it is important to note that EZH2 is dispensable for the B cell extrafollicular response^7, 8^, so that the observed organoid *Ezh2* knockout or EZH2 inhibitor effect is a true GC phenotype. To address our EZH2 mechanism question we were prompted to use this system to robustly demonstrate the critical requirement of EZH2 for cell cycle progression, which is difficult in vivo since natural GC are heterogeneous and composed of cells continuously recycling between light zone and dark zone and manifest variable degrees of proliferative potential. Future research on GC mimetic organoids will take advantage of the tunable design parameters of this system to recapitulate more complex features of the GC reaction. Yet overall our data show that this 3D B cell follicular organoid design represents a reliable system to study GC mechanistic questions in a tractable manner, likely to be broadly useful for immunology and lymphoma research.

Finally, it is important to note that the mechanism by which EZH2 controls GC B cell proliferation through regulation of *CDKN1A* is also relevant to the malignant lymphomas that arise from GC B cells. Proliferation arrest is the dominant and most obvious effect of EZH2 inhibitors or EZH2 shRNA in GCB type DLBCL cells, regardless of whether EZH2 is wild type or affected by gain of function mutation^7^. This effect was strongly and specifically linked to *CDKN1A* derepression by EZH2 lymphoma cells. Our current data indicate that this *CDKN1A* repression dependence of lymphoma cells is inherited from and based on the natural physiological function of EZH2 in GC B cells and is not an artifact of EZH2 somatic mutation or the malignant phenotype. EZH2 inhibitors are active against DLBCL in human patients and these drugs are currently in phase II clinical trials. Our data suggest that CDKN1A loss of function, or perhaps deregulation of CDK4/6 or CDK2 could result in acquired resistance to EZH2 inhibitors and should be considered in patients who break through EZH2 inhibitor therapy.

## Methods

### Mouse models

Conditional *Ezh2* knockout mice (*loxP*-flanked Ezh2 allele, *Ezh2* ^*fl/fl*^, C57BL/6) were a generous gift of Dr. Alexander Tarakhovsky, The Rockefeller University^10^. By crossing *Ezh2* ^*fl/fl*^ with the transgenic Cγ1-cre strain (C57BL/6) we generated heterozygous *Ezh2* ^*fl/WT*^ mice, which were crossed to obtain *Ezh2* ^*fl/fl*^ mice. These mice were crossed to *Cdkn1a* ^−/−^ (B6129SF2/J) to obtain *Ezh2* ^*fl/WT*^;Cγ1-cre^+^;*Cdkn1a* ^-/+^ heterozygous, which were further crossed to yield *Ezh2* ^*fl/fl*^;Cγ1-cre^+^;*Cdkn1a* ^−/−^ double knockout (mixed B6;129 background). As control groups, we used *Ezh2* ^*fl/fl*^;Cγ1-cre negative and the single knockouts *Ezh2* ^*fl/fl*^;Cγ1-cre^+^ and *Cdkn1a* ^−/−^ littermates (mixed B6;129 background). The following strains were purchased from The Jackson Laboratory: Cγ1-cre (010611), *Cdkn1a* ^−/−^ (003263), *E2f1* ^−/−^ (002785), B6129SF2/J (101045), and C57BL6 (000664).

Animal care was in strict compliance with institutional guidelines established by the Weill Cornell Medical College, the Memorial Sloan-Kettering Cancer Center, the Guide for the Care and Use of Laboratory Animals (National Academy of Sciences 1996)^37^, and the Association for Assessment and Accreditation of Laboratory Animal Care International.

### Germinal center assessment in mice

The Research Animal Resource Center of the Weill Cornell Medical College of Medicine approved all mouse procedures. Age- and sex-matched mice were immunized intraperitoneally at 8–12 weeks of age with either 0.5 ml of a 2% sheep red blood cell (SRBC, Cocalico Biologicals) suspension in PBS or 100 µg of highly substituted NP-KLH (NP-32 Keyhole Limpet Hemocyanin, Biosearch Technologies) in alum (Thermo Scientific), or 100 µg of highly substituted NP-CGG (NP-28 Chicken Gamma Globulin, Biosearch Technologies), and were killed after 8–10 or 14–60 days, respectively.

For GSK503 experiments: mice were randomized before treatment. Drug or vehicle (20% captisol) was injected intraperitoneally starting the day after induction of GC by SRBC and administered daily at a concentration of 150 mg/kg/day for 9 consecutive days after which the mice were killed (day 10).

### 3D B cell follicular organoid

For B cell purification spleens were freshly obtained from C57BL6, B6129SF2/J, *Cdkn1a* ^−/−^, *Ezh2* ^*fl/fl*^;Cγ1-cre, *Ezh2* ^*fl/fl*^;Cγ1-cre;*Cdkn1a* ^−/−^ double knockout and *Ezh2* ^*fl/fl*^ mice. Splenocytes were isolated by a combination of mechanical and gradient separation methods (Fico/Lite-LM, Atlanta Biologicals). B cells were obtained from splenocytes through negative selection using EasySep^TM^ Mouse B cell Isolation Kit (Stem Cell Technologies, 19854) in accordance with manufacturer’s protocol (yield ~ 90% B220^+^). 40LB cells, that express CD40L and produce BAFF, were generated as reported earlier by Nojima et al.^14^ and cultured in DMEM media with 10% FBS and penicillin G/streptomycin. 40LB cells were mitotically inhibited through incubation in cell culture complete medium containing 0.01 mg/ml Mitomycin C (Sigma-Aldrich, MO503) at 37 °C for 55 min and were rinsed twice with 10 ml of PBS before the encapsulation.

For organoid fabrication gelatin stock solution was freshly prepared by mixing gelatin powder (Sigma-Aldrich) in RPMI 1640 medium followed by sterilization using syringe filter. Cells were mixed with warmed 5% gelatin stock solution and diluted accordingly using cell culture medium. Silicate nanoparticles (SiNP) with 25–30 nm in diameter and 1 nm in thickness were obtained from Southern Clay Products Inc., USA. 3% hydrogel SiNP suspension was freshly prepared before the encapsulation procedure by mixing SiNP powder with deionized water and vortexing the resulting solution, followed by filtration through 0.22 µm syringe filters immediately before use. Organoids were fabricated in 96-well plates by first adding 10 µl of 3% hydrogel SiNP followed by injecting 10 µl cell-containing gelatin solution into the initial SiNP droplet, and then mixing the entire hydrogel through repeated pipetting. Each organoid contained 50,000 B cells and 80,000 40LB cells. Organoids were cured for ~ 10 min before the addition of RPMI media with 10% FBS and penicillin G/streptomycin, containing 50 ng/ml murine recombinant IL-4 (R&D, 404-ML) and were incubated at 37 °C with 5% CO_2_. The medium was replaced every 3 days. When organoids were treated with GSK343 or GSK669, the drugs were dissolved in DMSO such that the final concentration of DMSO in culture was 0.02%.

2D B cell cultures also contained gelatin, 40LB cells and IL-4. The only difference with 3D organoid culture was the hydrogel SiNP 3D matrix.

### Flow cytometry analysis

Single-cell suspensions from mouse spleens and 3D B cell follicular organoids were stained using the following fluorescent-labeled anti-mouse antibodies: from eBioscience: PE-Cy7 anti-B220 (25-0452, dilution 1:750), APC anti-CD38 (17-0381, dilution 1:750), PE anti-CXCR4 (12-9991, dilution 1:400), APC anti-CD4 (17-0041, dilution 1:750), FITC anti-PD-1 (11-9985, dilution 1:400); from BD Biosciences: APC anti-B220 (553092, dilution 1:750), PE and PE-Cy7 anti-FAS (554258 and 557653, dilution 1:750), FITC anti-GL7 (553666, dilution 1:750), FITC anti-IgM (553437, dilution 1:500), PE-Cy7 anti-CD86 (560582, dilution 1:400), AlexaFluor488 anti-EZH2 (562479, dilution 1:50), AlexaFluor488 IgG1κ isotype control (557721, dilution 1:100), V450 anti-BrdU (560810, dilution 1:500); from Cell Signaling: AlexaFluor674 anti-H3K27me3 (12158, dilution 1:300), AlexaFluor674 IgG isotype control (2985, dilution 1:300); from BioLegend: PerCP-Cy5.5 anti-GL7 (144610, dilution 1:750), PerCP anti-IgD (405736, dilution 1:750), PE-Cy7 anti-CD138 (142514, dilution 1:500), PE anti-BCL6 (648304, dilution 1:20), PE IgG1κ isotype control (400112, dilution 1:1,600); from Biosearch Technologies: PE NP (N-5070-1, dilution 1:500). DAPI was used for the exclusion of dead cells. 7AAD was used to determine DNA content in cell cycle analysis. For CXCR5 staining, cells were incubated with purified rat anti-mouse CXCR5 antibody (BD 551961, 2G8, dilution 1:100) followed by a biotin conjugated goat anti-rat antibody incubation (Jackson Immunoresearch, dilution 1:1000), followed by PE-Cy7 streptavidin (eBioscience 25-4317, dilution 1:1000). For internal markers, cells were fixed and permeabilized with BD Cytofix/Cytoperm Fixation/Permeabilization Solution Kit (BD Biosciences) and further permeabilized with cold Phosflow Perm Buffer III (BD Biosciences). To monitor cell divisions in organoids and 2D cultures, B cells were labeled with the proliferation dye eFluor670 (eBioscience) before the encapsulation procedure. PE conjugated Annexin V (BD Biosciences 556421, dilution 1:100) in Annexin V binding buffer (BD Biosciences) was used to identify apoptotic cells. Data were acquired on BD FACSCanto flow cytometer (BD Biosciences) and analyzed using FlowJo software package (TreeStar).

### Immunohistology

Mouse spleens were fixed in 4% formaldehyde and embedded in paraffin. Deparaffinized slides were antigen retrieved in citrate buffer pH 6.4 and endogenous peroxidase (HRP) activity was blocked by treating the sections with 3% hydrogen peroxide in methanol. Indirect immunohistochemistry was performed with antispecies-specific biotinylated secondary antibodies followed by avidin–horseradish peroxidase or avidin-AP, and developed by Vector Blue or DAB color substrates (Vector Laboratories). Sections were counterstained with hematoxylin if necessary. Biotin-conjugated PNA (Vector Laboratories B-1075, dilution 1:250) was used to identify germinal centers. The following antibodies were used: biotin-conjugated anti-B220 (Invitrogen RM2615, dilution 1:30), EZH2 (Cell Signaling 5246, dilution 1:200), Ki67 (Vector VP-K451, dilution 1:100), p105-Rb Ser780 (Bioss bs-1347R, dilution 1:100). Slides were scanned using a Zeiss Mirax Slide Scanner and photomicrographs were examined with Pannoramic Viewer software. ImageJ 1.44o software (NIH) was used to quantify germinal center areas.

### Immunoblotting

Lysates from splenocytes and DLBCL cells were prepared using 20 mM Tris, pH 8, 135 mM NaCl, 1% NP-40, 10% glycerol, 1 mM PMSF, and complete protease inhibitor cocktail (Roche) lysis buffer. Protein lysates were resolved by SDS–PAGE, transferred to PVDF membrane, and probed with the indicated primary antibodies: EZH2 (Active Motif 39933, dilution 1:1000), H3K27me3 (Millipore 07-449 and 17-622, dilution 1:20,000), pan-Histone 3 (Millipore 07-690, dilution 1:50,000), βActin (Sigma A5441, dilution 1:5,000), p21 (Cell Signaling 2947 for human cells, dilution 1:500, and Millipore 05-345 for murine cells, dilution 1:500), E2F1 (Cell Signaling 3742 for human cells, dilution 1:1000, and Abcam 179445 for murine cells, dilution 1:500), E2F2 (Abcam 138515, dilution 1:1000), p105-Rb Ser780 (Cell Signaling 8180, dilution 1:500), GAPDH (Santa Cruz 25778, dilution 1:20,000). Membranes were then incubated with a peroxidase-conjugated correspondent secondary antibody and detected using enhanced chemiluminescence. Densitometry values were obtained by using ImageJ 1.44o software (NIH).

### Cell lines

The DLBCL cell lines OCI-Ly1, OCI-Ly7 and OCI-Ly18 were grown in Iscove’s medium supplemented with 10% FBS and penicillin G/streptomycin; WSU-DLCL2, Farage, Pfeiffer and OCI-Ly19 in RPMI medium supplemented with 10% FBS, penicillin G/streptomycin, l-glutamine, and HEPES. OCI-Ly1, OCI-Ly7, and OCI-Ly18 were obtained from Ontario Cancer Institute in June 2011. Farage and Pfeiffer were obtained from ATCC, and WSU-DLCL2 and OCI-Ly19 from DSMZ in May 2011. All the cell lines were recently authenticated by Biosynthesis using their STR Profiling and Comparison Analysis Service, between October and December 2016. These cell lines have also been routinely tested for mycoplasma contamination in the laboratory.

### B cell purification

Human B cell populations were affinity-purified from de-identified human tonsillectomy specimens using standard protocols^28^ with approval from the Human Research Protections Programs, Division of Research Integrity of the Weill Cornell Medical College, in accordance with the Declaration of Helsinki. naive B and GC B cell purity ( > 90%) was determined by flow cytometry analysis of surface IgD (BD Biosciences 555778), CD77 (BioRad MCA579) and CD38 (BD Biosciences 340439).

Murine B cell populations from immunized mice and organoid and 2D cultures were purified by FACS using FAS^−^ GL7^−^ IgD^+^ B220^+^ splenocytes as naive B cells, and FAS^+^ GL7^+^ B220^+^ splenocytes as GC B cells.

### ELISA

Murine serum samples were collected one day before NP-KLH immunization and 14, 21, 26, 35 and 60 days post NP-KLH, and immunoglobulin levels were analyzed by ELISA. Sera were tested for the binding of NP-specific IgG1, IgG2b and IgA antibodies (SouthernBiotech, dilution 1:500) to NP_4_-BSA and NP_30_-BSA coated plates (Biosearch Technologies). Optical density (OD) at 405 nm was measured in a plate reader (BioTek) and the absorbance ratio was calculated by dividing the mean OD in NP_4_-BSA coated wells by the mean OD in NP_30_-BSA coated wells.

### ELISPOT

Murine bone marrow cells were collected 60 days after NP-KLH immunization, and IgG1 production was analyzed by ELISPOT. ELISPOT plates (Millipore, MAHAS4510) were activated by incubation with 35% ethanol. Cells were tested for the binding of NP-specific IgG1 antibody (SouthernBiotech, dilution 1:500) to NP_4_-BSA and NP_30_-BSA coated plates (Biosearch Technologies). The plates were scanned and number of spots analyzed by ZellNet Consulting Inc., NJ. The ratio of the number of spots was calculated by dividing the mean # spots in NP_4_-BSA coated wells by the mean # spots in NP_30_-BSA coated wells.

### Plasmids, shRNAs and virus production

EZH2 complementary DNA (cDNA) was cloned into pRetroX-ZsGreen vector (Clontech). Retroviruses were produced by transfection of amphotropic 293T cells with appropriate plasmids and FuGENE 6 Transfection reagent (Roche). E2F1 and E2F2 shRNAs were delivered by lentivirus infection, which were produced by transfection of 293T cells with the vector pLKO.1 with puromycin resistance. Infected cells were selected by puromycin treatment (1 µg/mL). Mature antisense sequences of shRNA were: shE2F1: 5′-TAACTGCACTTTCGGCCCTTT-3′, shE2F2: 5′-GCCTATGTGACTTACCAGGAT-3′.

### ChIP and qPCR

ChIP was performed as previously described^28^. Briefly, 10^8^ cells were fixed with 1% formaldehyde, lysed, and sonicated (Branson Sonicator; Branson) leading to a DNA average size of 200 bp. Five µg of antibodies anti-EZH2 (Active Motif 39901), H3K27me3 (Abcam 6002), E2F1 (Cell Signaling 3742) or control IgG (Millipore 12-370, 12-371) were added to the precleared sample and incubated overnight at 4 °C. The complexes were purified using protein-A beads (Roche) followed by elution from the beads and decrosslinking. DNA was purified using PCR purification columns (QIAGEN) and was amplified by real-time quantitative PCR using SyberGreen (Applied Biosystems) on 7900HT Fast Real-Time PCR System (Applied Biosystems).

### RT-qPCR

RNA was prepared using Trizol extraction (Invitrogen). cDNA was prepared using cDNA synthesis kit (Thermo Scientific) and detected by fast SyberGreen (Applied Biosystems) on 7900HT Fast Real-Time PCR System (Applied Biosystems). We normalized gene expression to HPRT1 or GAPDH and expressed values relative to control using the ^ΔΔ^CT method. Results were represented as fold expression with the standard deviation for 2 series of triplicates.

Primers used for ChIP and cDNA qPCR are shown in Supplementary Table 1.

### mRNA-seq library preparation and sequencing processing

RNA-seq libraries were prepared using the Illumina TruSeq RNA sample kits, according to the manufacturer. Libraries were validated using the Agilent Technologies 2100 Bioanalyzer and Quant-iT™ dsDNA HS Assay (Life Technologies), and 2000–2200 hours sequenced on HiSeq2000 sequencer mRNA-seq, single read, 50 bp.

### mRNA-seq analysis

RNA sequencing results were aligned to mm10 using STAR^31^ and annotated to RefSeq using the R subread package^32^. Differentially expressed genes were identified using the EdgeR package GLM^33^ with thresholds of fold-change > 1.5 and *P* < 0.01, adjusted for multiple testing using Benjamini-Hochberg correction. Principal component analysis was performed on centered, log-transformed FPKM values using the prcomp() function within R framework. Principal component genes were identified as those 2s.d. above or below the respective mean loading factor. Unrooted phylogenetic tree was constructed using neighbor-joining tree estimation within the ape R package^34^. Gene set enrichment analysis was performed using the GSEA algorithm, as described in ref. ^35^, using genes differentially expressed between naive and germinal center B cells.

### Bone marrow transplantation

Murine bone marrow transplantation assays were performed as described previously^36^. Briefly, bone marrow cells from 6-8 week old *E2f1* ^−/−^ or B6129SF2/J control male donors were harvested and cells were transduced with viral supernatants containing either pRetroX-IRES-ZsGreen1 empty vector or pRetroX-EZH2-IRES-ZsGreen1. The transduction efficiency was between 30 and 40%, and was similar between the experimental groups. One million bone marrow cells of each type were injected into the tail veins of lethally irradiated (900 rad) female B6129SF2/J mice.

### Immunoglobulin SHM analysis

Immunoglobulin mutation analysis was performed on genomic DNA of GCB and naive B cells sorted from 3 immunized WT mice, purified naive B cells from 3 WT spleens used for 3D organoid cultures, and GCBs sorted from 3D organoid after 6 days of culture. IgH and Igλ regions were PCR amplified using primers that anneal to the framework region of the most abundant families of Ig rearrangements, as described previously^38–41^. PCR products were cleaned-up using the MinElute PCR Purification kit (QIAGEN) and subsequently were purified on a gel using the Gel Extraction kit (QIAGEN). Sequencing libraries were constructed from the purified PCR product by using Illumina TruSeq DNA Sample Preparation Kit v2 (Illumina). Each sample was tagged with a unique index and sequenced on the Illumina MiSeq platform producing 2 × 151bp paired-end reads. Paired-end sequence reads were mapped against the Mus musculus primary assembly GRCm38 using aligner Star 2.4.0^31^. A pileup of the resulting sorted bam files was made in samtools for each targeted region was made filtering by quality score > 20. A list of all single nucleotide polymorphisms was made using VarScan 2.3.4^42^ on each base with minimum read depth of 10 reads and tabulated per targeted region into bed-files. These bed-files were then used to create the mutation rate per kilobase for each gene by dividing the average missense or indels per base by the average coverage of sequenced read and multiplying by 1000 to obtain the missense or indel rate per kilobase. The average mutation rate per kilobase in GC B cells was normalized to the average in naive B cells.

Primers used for immunoglobulin mutation analysis are shown in Supplementary Table 2.

### EZH2 small molecule inhibitors

GSK343 was synthesized, as described in ref. ^43^. GSK126 was synthesized, as described in ref. ^44^, and GSK503, as described in ref. ^7^.

### Statistics

Given that the sample sizes in the two groups that were compared were equal, with similar variance and assuming they follow a normal distribution, pairwise comparisons of cell numbers and percentages, MFI, qPCR, gene expression values, mutation rate and qChIP were assessed using unpaired parametric Students *t* test. Two-tailed *P* values are indicated in the figure legends. For animal studies, the sample size was estimated based on previous experiments^7, 30^, selecting the optimum number of animals needed to attain statistical significance of an alpha level < 0.05 with 90% probability. Gene set enrichment was assessed using the GSEA algorithm, a computational method based on the Kolmogorov–Smirnov test.

### Data availability

Sequence data that support the findings of this study have been deposited in GEO with the primary accession codes GSE95491 and GSE45982.

## Electronic supplementary material

Supplementary information

Peer review file
