# Supplementary material for: EZH2 enables germinal centre formation through epigenetic silencing of CDKN1A and an Rb-E2F1 feedback loop
Source: Nat Commun. 2017 Oct 12;8:877. doi: 10.1038/s41467-017-01029-x (PMC5638898; doi:10.1038/s41467-017-01029-x)
Supplement: Supplementary file 1 — Supplementary information [file 41467_2017_1029_MOESM1_ESM.pdf]

SUPPLEMENTARY FIGURES

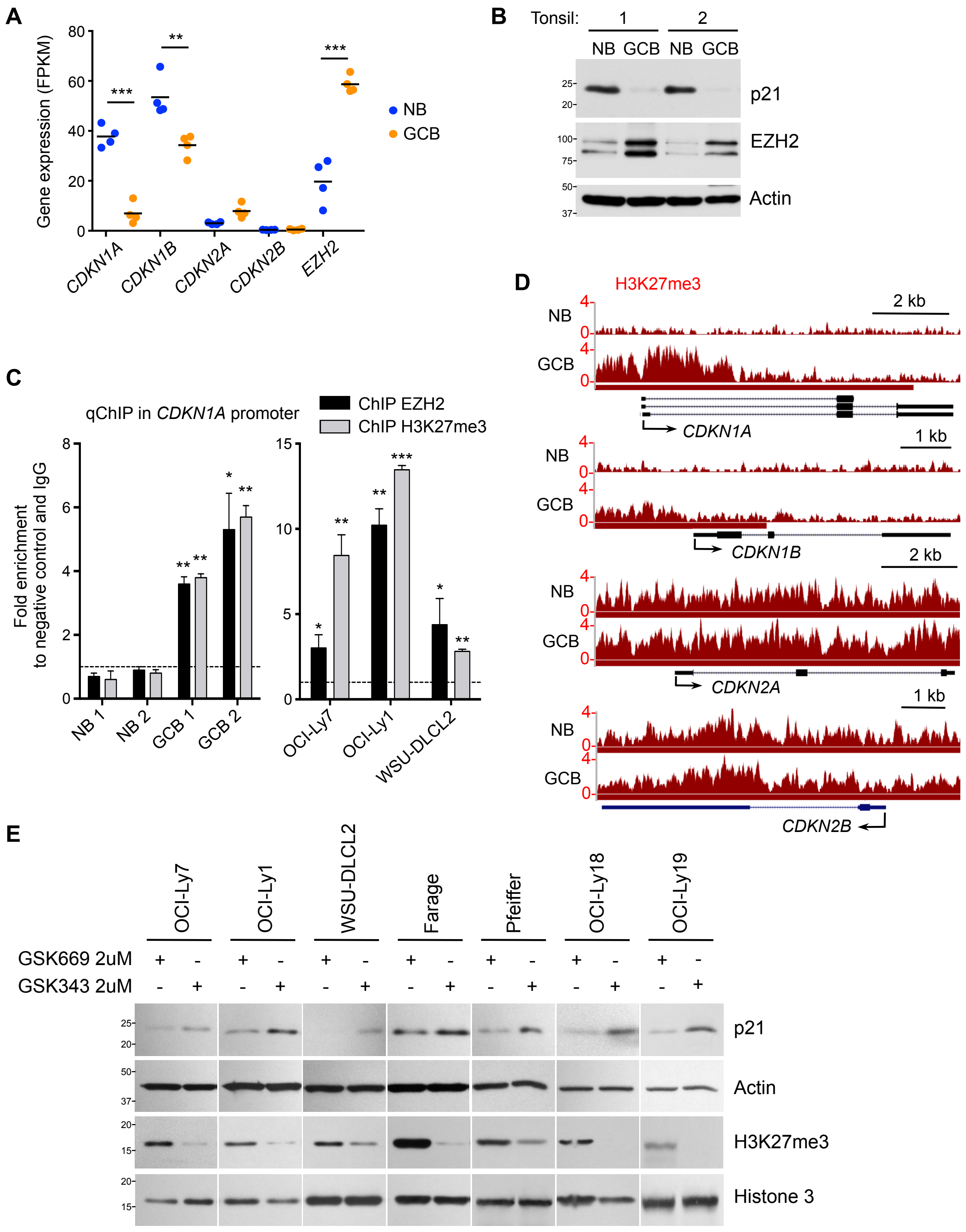

Supplementary Figure 1. CDKN1A is down-regulated in GC B cells and is a direct target of EZH2 (**A**) Expression level in FPKM of the indicated genes in naïve B cells (NB) and germinal center B cells (GCB) from 4 human tonsils. Horizontal black lines represent mean. t test, **p<0.01, ***p<0.001. (**B**)

Immunoblotting of whole cell lysates from NB and GC B cells from two human tonsil samples. Actin was used as loading control. The western blot shown is representative of a total of 5 tonsils analyzed. Numbers on the left indicate molecular weight in kDa. (**C**) EZH2 and H3K27me3 qChIP in NB and GC B cells from two human tonsils (left) and the indicated cell lines (right). qPCR was performed using primers targeting the promoter of *CDKN1A*. As negative control qPCR was performed using primers for a region in chromosome 6 where no H3K27me3 enrichment was found by ChIP-seq read density in GC B cells. EZH2 and H3K27me3 fold enrichment was normalized to the enrichment at the negative control region and further normalized to IgG qChIP. Values are shown as mean of technical triplicates ± SD. t test vs. IgG ChIP, *p<0.05, **p<0.01, ***p<0.001. Two biological replicates in tonsils are shown. The cell line ChIPs shown are representative of 4 independent experiments. (**D**) *CDKN1A, CDKN1B, CDKN2A* and *CDKN2B* gene loci showing H3K27me3 ChIP-seq read density in NB and GC B cells. Red bar represents H3K27me3 domain. (**E**) Representative immunoblotting of whole cell lysates from the indicated cell lines treated with 2μM EZH2 inhibitor GSK343 or the control compound GSK669 for 72 hours. Actin and Histone 3 were used as loading controls. The experiment was repeated 3 to 4 times with similar results.

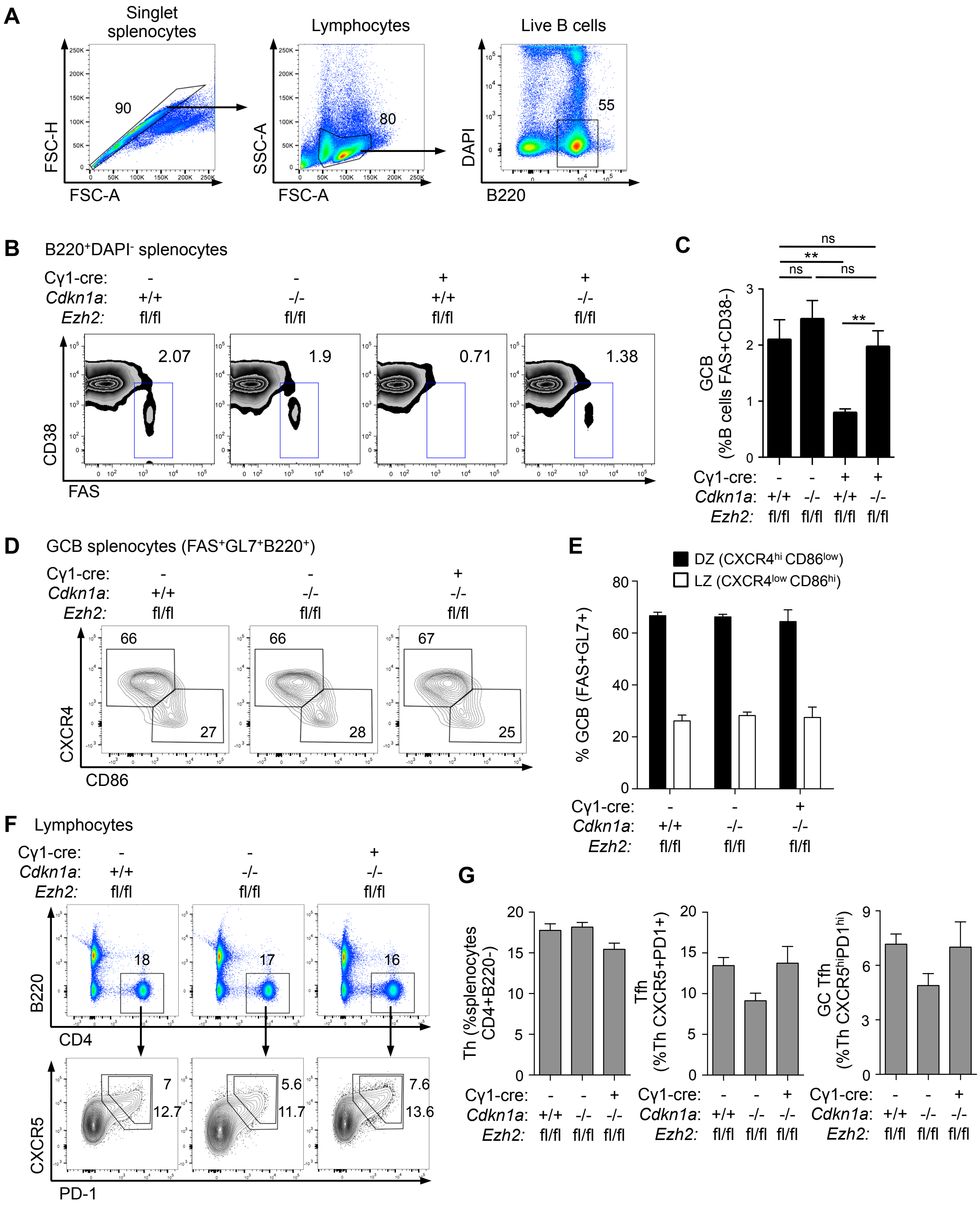
A
Singlet splenocytes
Lymphocytes
Live B cells
90
80
55
FSC-H
FSC-A
SSC-A
FSC-A
DAPI
B220
B
B220+DAPI- splenocytes
Cγ1-cre: - - + +
Cdkn1a: +/+ -/- +/+ -/-
Ezh2: fl/fl fl/fl fl/fl fl/fl
2.07
1.9
0.71
1.38
CD38
FAS
C
ns
**
ns
ns
**
GCB (%B cells FAS+CD38-)
Cγ1-cre: - - + +
Cdkn1a: +/+ -/- +/+ -/-
Ezh2: fl/fl fl/fl fl/fl fl/fl
D
GCB splenocytes (FAS+GL7+B220+)
Cγ1-cre: - - +
Cdkn1a: +/+ -/- -/-
Ezh2: fl/fl fl/fl fl/fl
66
27
66
28
67
25
CXCR4
CD86
E
DZ (CXCR4hi CD86low)
LZ (CXCR4low CD86hi)
% GCB (FAS+GL7+)
Cγ1-cre: - - +
Cdkn1a: +/+ -/- -/-
Ezh2: fl/fl fl/fl fl/fl
F
Lymphocytes
Cγ1-cre: - - +
Cdkn1a: +/+ -/- -/-
Ezh2: fl/fl fl/fl fl/fl
18
17
16
B220
CD4
7
12.7
5.6
11.7
7.6
13.6
CXCR5
PD-1
G
Th (%splenocytes CD4+B220-)
Tfh (%Th CXCR5+PD1+)
GC Tfh (%Th CXCR5hiPD1hi)
Cγ1-cre: - - +
Cdkn1a: +/+ -/- -/-
Ezh2: fl/fl fl/fl fl/fl

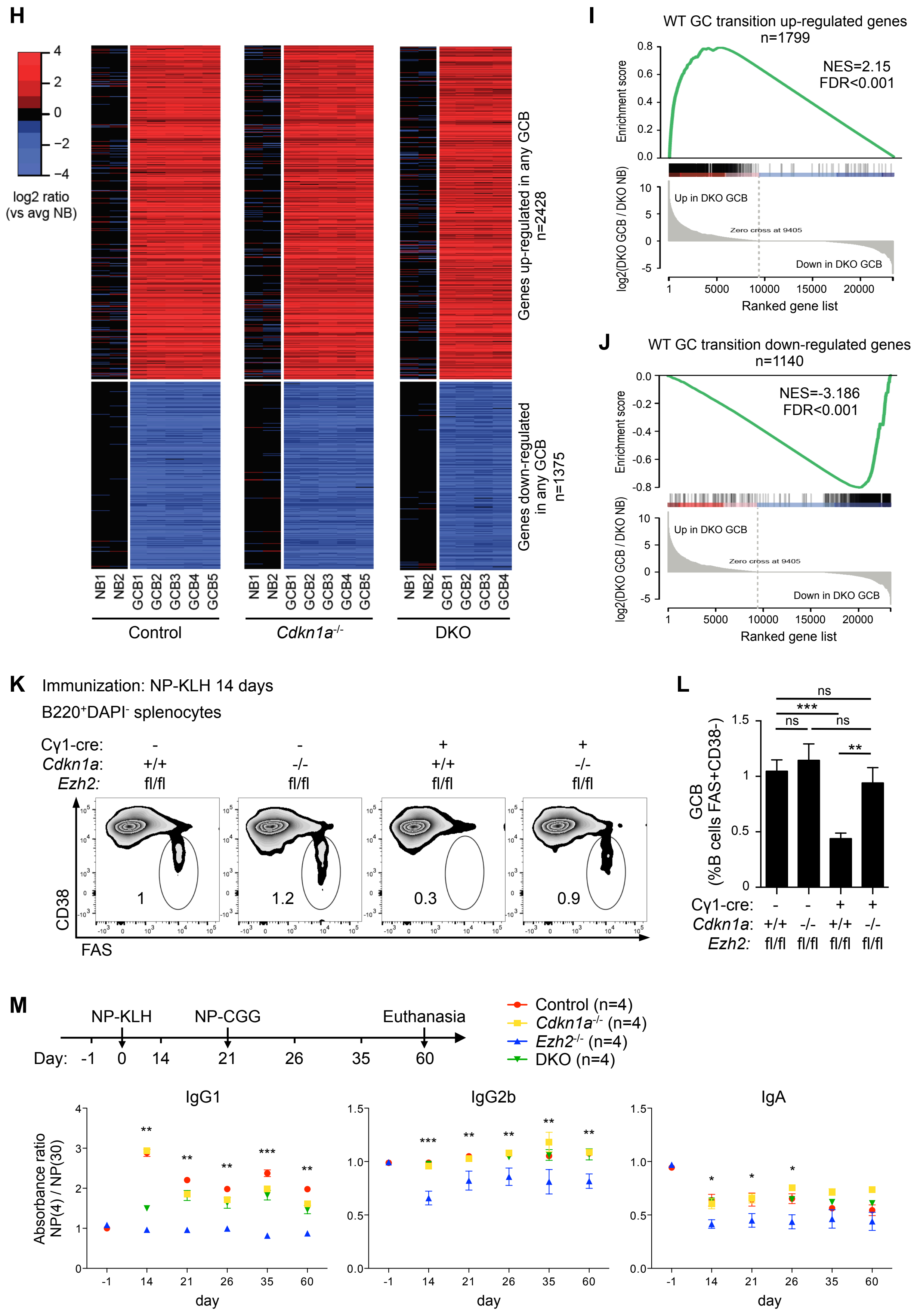
H
log2 ratio (vs avg NB)
Genes up-regulated in any GCB n=2428
Genes down-regulated in any GCB n=1375
NB1 NB2 GCB1 GCB2 GCB3 GCB4 GCB5
Control
Cdkn1a-/-
DKO
I
WT GC transition up-regulated genes n=1799
NES=2.15 FDR<0.001
Enrichment score
log2(DKO GCB / DKO NB)
Up in DKO GCB
Zero cross at 9405
Down in DKO GCB
Ranked gene list
J
WT GC transition down-regulated genes n=1140
NES=-3.186 FDR<0.001
K
Immunization: NP-KLH 14 days
B220+DAPI- splenocytes
Cγ1-cre: - - + +
Cdkn1a: +/+ -/- +/+ -/-
Ezh2: fl/fl fl/fl fl/fl fl/fl
1
1.2
0.3
0.9
CD38
FAS
L
GCB (%B cells FAS+CD38-)
ns
***
ns
ns
**
M
NP-KLH
NP-CGG
Euthanasia
Day: -1 0 14 21 26 35 60
Control (n=4)
Cdkn1a-/- (n=4)
Ezh2-/- (n=4)
DKO (n=4)
IgG1
IgG2b
IgA
Absorbance ratio NP(4) / NP(30)
day

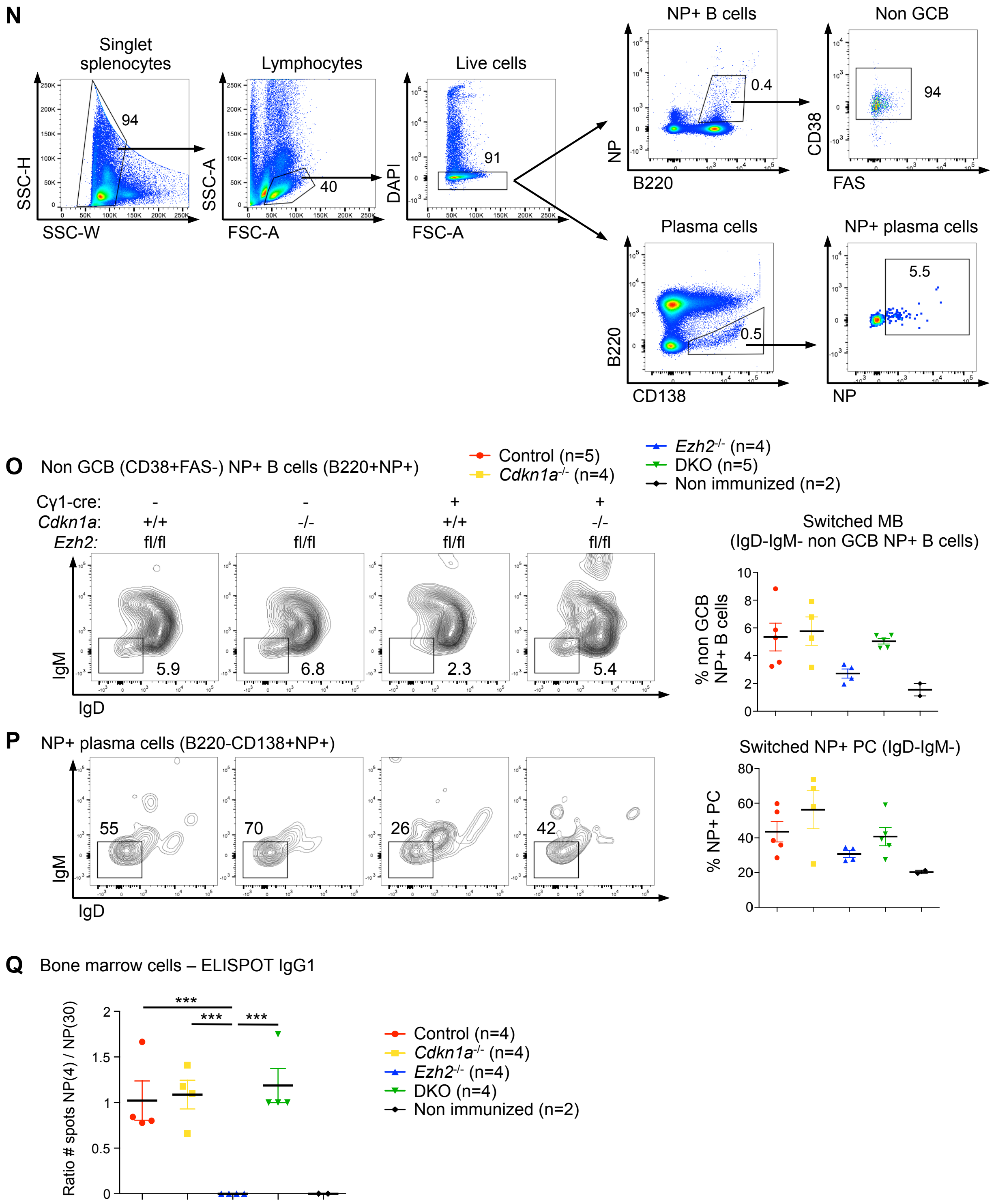

Supplementary Figure 2, related to figure 1. **In vivo depletion of *Cdkn1a* rescues GC formation in *Ezh2*$^{-/-}$ mice**

(**A**) Flow cytometry plots of one representative mouse spleen showing the gating strategy used in Figures 1A, 1G, 2A, 2G, 2H, 7B, and Supplementary Figures 2B, 2K and 3A. (**B-C**) Splenocytes from *Ezh2*$^{fl/fl}$, *Cdkn1a*$^{-/-}$, *Ezh2*$^{fl/fl}$;Cγ1-cre and *Ezh2*$^{fl/fl}$;Cγ1-cre;*Cdkn1a*$^{-/-}$ littermate mice from Figure 1 were stained to identify GC B cells with alternative markers (FAS+CD38-) within B cells (B220+). (**B**) Flow cytometry plot

of one representative mouse spleen per group. The gated area shows the percentage of GC B cells (FAS+CD38-) within live B cells (B220+DAPI-). (**C**) Average of GC B populations of each group of mice quantified by flow cytometry as in (B) (n=7 per group). t test, **p<0.01. (**D-G**) *Ezh2*$^{fl/fl}$, *Cdkn1a*$^{-/-}$ and *Ezh2*$^{fl/fl}$;Cγ1-cre;*Cdkn1a*$^{-/-}$ mice were immunized with SRBC and sacrificed 10 days later. (**D**) Flow cytometry plot of one representative mouse spleen per group. The gated areas show the percentage of light zone GC B cells (CXCR4^{low}CD86^{hi}) and dark zone GC B cells (CXCR4^{hi}CD86^{low}) within live GC B cells (GL7+FAS+B220+DAPI-). (**E**) Average of light zone (LZ) and dark zone (DZ) GC B populations of each group of mice quantified by flow cytometry as in (D) (n=5 mice per group). (**F**) Flow cytometry plot of one representative mouse spleen per group. The gated areas on the bottom show the percentage of Tfh (CXCR5+PD-1+) and GC Tfh (CXCR5^{hi}PD-1^{hi}) within Th splenocytes (CD4+B220-, showed on the top panel). (**G**) Average of Th, Tfh and GC Tfh populations of each group of mice quantified by flow cytometry as in (F) (n=5 mice per group). (**H**) Heat maps of gene expression level of GC B cells from *Ezh2*$^{fl/fl}$ ("control", n=5), *Cdkn1a*$^{-/-}$ (n=5) and *Ezh2*$^{fl/fl}$;Cγ1-cre;*Cdkn1a*$^{-/-}$ double knockout mice ("DKO", n=4) immunized with SRBC for 10 days, represented as log2 ratio relative to mean naïve B cells (n=2 per group). (**I-J**) GSEA showing enrichment of murine WT GC B cell signature genes for (**I**) up- and (**J**) down-regulation of gene expression in *Ezh2*$^{fl/fl}$;Cγ1-cre;*Cdkn1a*$^{-/-}$ DKO GC B cells versus NB. (**K-L**) *Ezh2*$^{fl/fl}$, *Cdkn1a*$^{-/-}$, *Ezh2*$^{fl/fl}$;Cγ1-cre and *Ezh2*$^{fl/fl}$;Cγ1-cre;*Cdkn1a*$^{-/-}$ littermate mice (n=5 per group) were immunized with NP-KLH and animals were sacrificed 14 days later. (**K**) Representative flow cytometry plot of splenic GC cells (FAS+CD38-B220+). (**L**) Average of GC B populations of each group of mice (n=5 per group) quantified by flow cytometry as in (K). t test, **p<0.01, ***p<0.001. (**M**) Four *Ezh2*$^{fl/fl}$ ("control"), 4 *Cdkn1a*$^{-/-}$, 4 *Ezh2*$^{fl/fl}$;Cγ1-cre ("*Ezh2*$^{-/-}$") and 4 *Ezh2*$^{fl/fl}$;Cγ1-cre;*Cdkn1a*$^{-/-}$ DKO mice were immunized and serum samples were collected as shown in the scheme. Anti-NP antibodies (IgG1, IgG2b and IgA) were measured in the sera by ELISA. The graphs show means ± SEM. t test DKO vs. *Ezh2*$^{-/-}$, *p<0.05, **p<0.01, ***p<0.001. (**N**) Flow cytometry plots of one representative mouse spleen showing the gating strategy used in Supplementary Figures 2O-P. (**O-P**) Splenocytes from 5 *Ezh2*$^{fl/fl}$ ("control"), 4 *Cdkn1a*$^{-/-}$, 4 *Ezh2*$^{fl/fl}$;Cγ1-cre ("*Ezh2*$^{-/-}$") and 5 *Ezh2*$^{fl/fl}$;Cγ1-cre;*Cdkn1a*$^{-/-}$ DKO mice immunized with NP-KLH, boosted with NP-CGG and euthanized 60 days later as shown in (M), and splenocytes from 2 non immunized control mice were stained to identify class switched (IgD-IgM-) within (**O**) NP positive memory B cells (MB) (CD38+FAS-NP+B220+), and within (**P**) NP positive plasma cells (PC) (NP+CD138+B220-), as shown in (N). The average ± SEM of class switched NP+ MB and PC populations of each group of mice quantified by flow cytometry is shown on the right. (**Q**) ELISPOT was performed to identify bone marrow IgG1+ NP specific cells from mice immunized as shown in (M). t test, ***p<0.001.

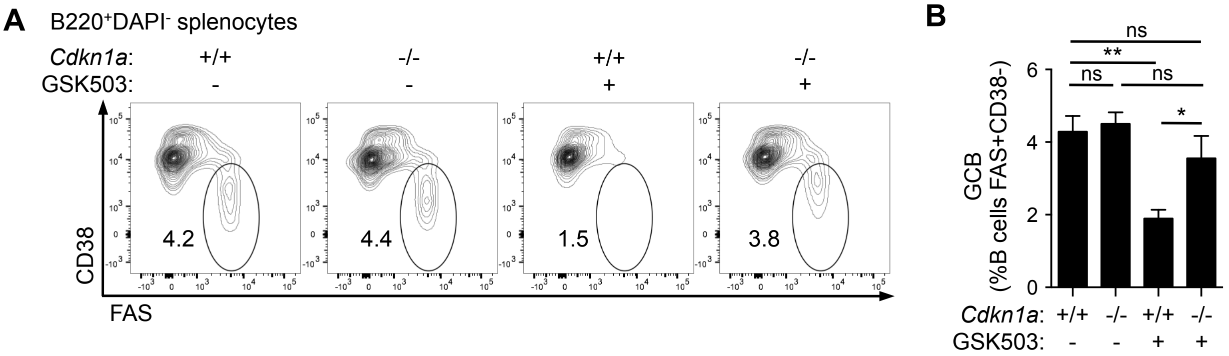

Supplementary Figure 3, related to figure 2. ***Cdkn1a*$^{-/-}$ GC rescue is linked to the histone methyltransferase function of EZH2**

Splenocytes from 5 *Cdkn1a*$^{+/+}$ and 5 *Cdkn1a*$^{-/-}$ mice treated with GSK503 or vehicle from Figure 2 were stained to identify GC B cells with alternative markers (FAS+CD38-) within B cells (B220+, see Supplementary Figure 2A for gating strategy). (**A**) Flow cytometry plot of one representative mouse spleen per group. The gated area shows the percentage of GC B cells (FAS+CD38-) within live B cells (B220+DAPI-). (**B**) Average of GC B populations of each group of mice quantified by flow cytometry as in (A). Values are shown as mean ± SEM (n=5 per group). t test, *p<0.05, **p<0.01.

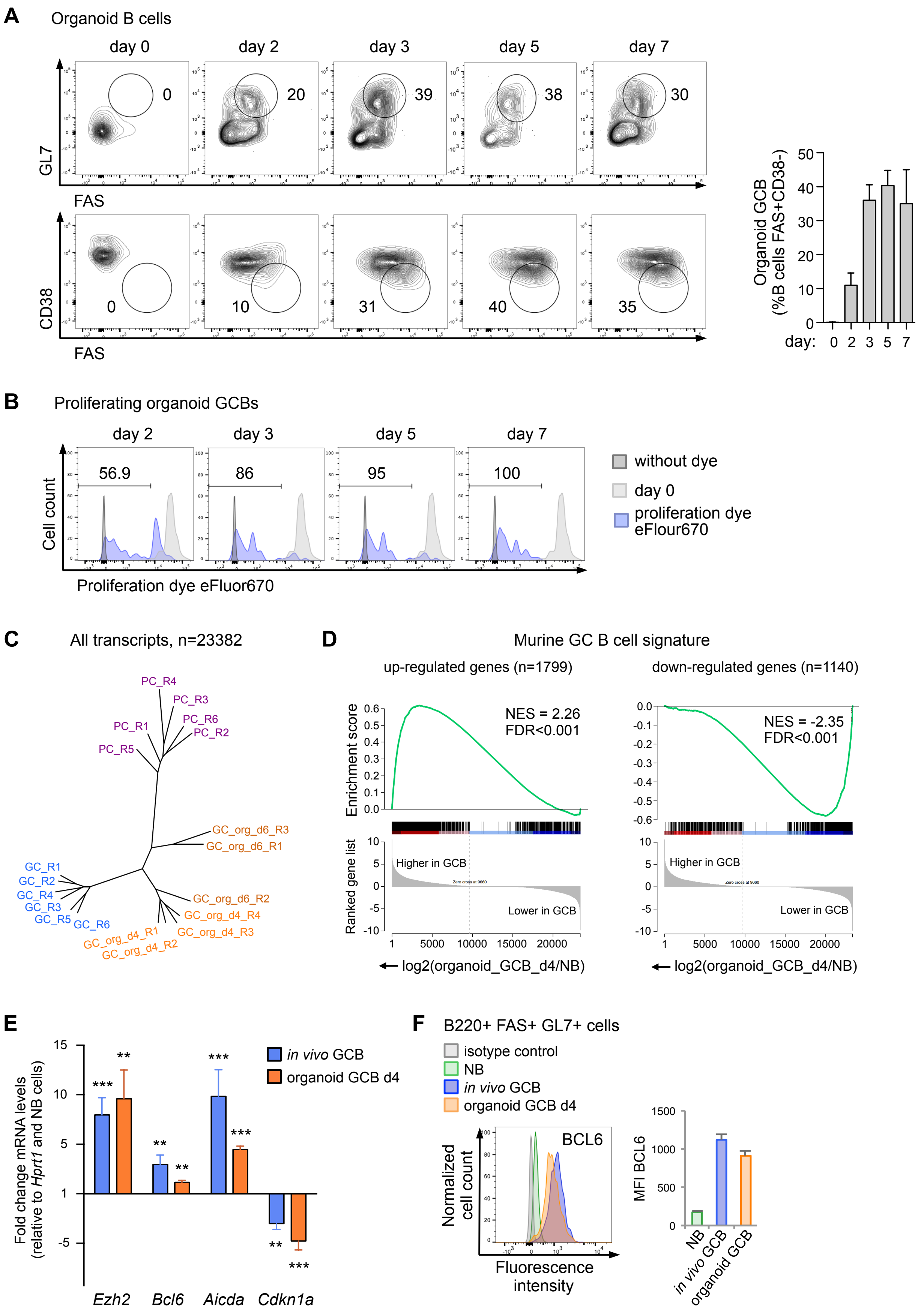
A
Organoid B cells
day 0
day 2
day 3
day 5
day 7
GL7
FAS
CD38
FAS
Organoid GCB
(%B cells FAS+CD38-)
day:
B
Proliferating organoid GCBs
day 2
day 3
day 5
day 7
56.9
86
95
100
Cell count
Proliferation dye eFluor670
without dye
day 0
proliferation dye eFlour670
C
All transcripts, n=23382
D
Murine GC B cell signature
up-regulated genes (n=1799)
down-regulated genes (n=1140)
NES = 2.26
FDR<0.001
NES = -2.35
FDR<0.001
Enrichment score
Ranked gene list
Higher in GCB
Lower in GCB
log2(organoid_GCB_d4/NB)
E
Fold change mRNA levels
(relative to Hprt1 and NB cells)
in vivo GCB
organoid GCB d4
Ezh2
Bcl6
Aicda
Cdkn1a
F
B220+ FAS+ GL7+ cells
isotype control
NB
in vivo GCB
organoid GCB d4
BCL6
Normalized cell count
Fluorescence intensity
MFI BCL6

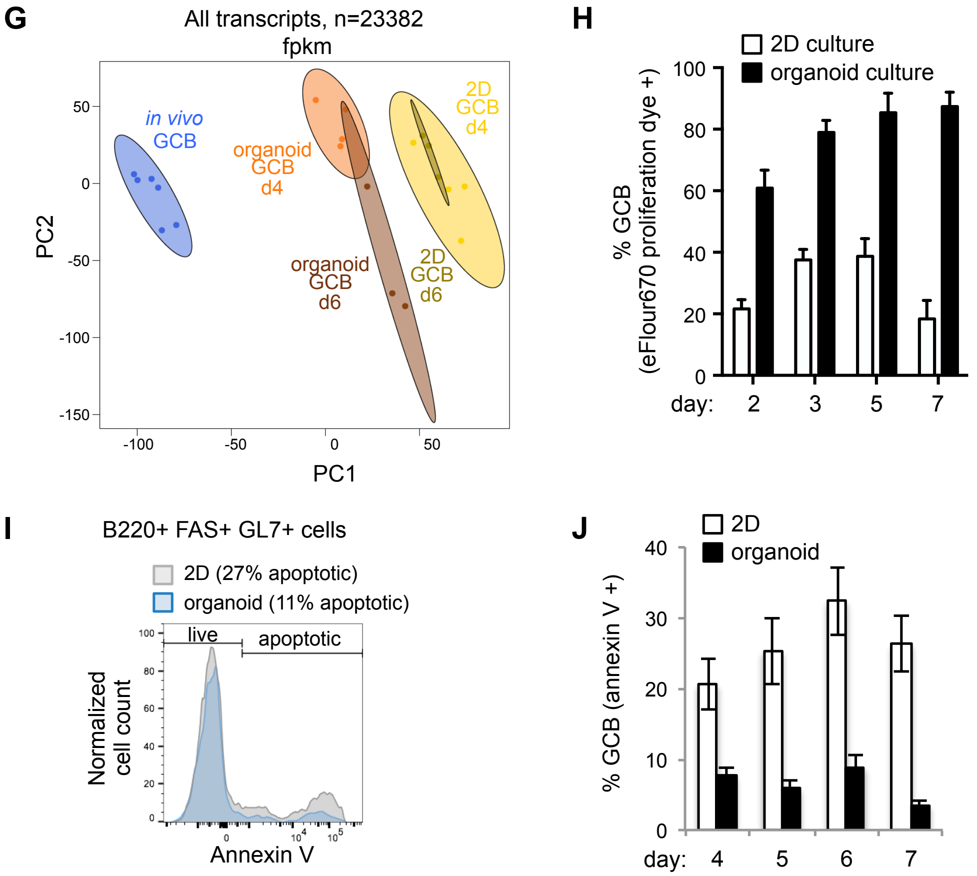

Supplementary Figure 4, related to figure 3**. Characterization of a 3D B cell follicular organoid to model the GC reaction**

(**A**) Flow cytometry plots of representative 3D B cell follicular organoid after 0, 2, 3, 5 and 7 days in culture. The gated areas show the percentage of organoid GC B cells (GL7+FAS+ on the top panel, and CD38-FAS+ on the bottom panel) within live B cells (B220+DAPI-). The histogram shows the average ± SEM of GC B populations (CD38-FAS+B220+) of organoids, quantified by flow cytometry (n=3). (**B**) Representative flow cytometry plots of fluorescence intensity of the proliferation dye eFluor670 in organoid GC B populations (GL7+FAS+B220+) cultured for 2, 3, 5 and 7 days. Results shown in (A) and (B) are representative of a total of 4 independent experiments. (**C**) Unrooted phylogenetic tree analysis was performed using RNA-seq profiles from FAS+GL7+B220+ organoid GC B cells after 4 and 6 days in culture (GC_org_d4, n=4, and GC_org_d6, n=3), FAS+GL7+B220+ GC B cells sorted from immunized mice (GC, n=6) and CD138+ plasma cells (PC, n=6). (**D**) GSEA showing enrichment of murine GC B cell signature genes for up- and down-regulation of gene expression in organoid GC B cells after 4 days in culture versus NB (culture day 0). (**E**) RT-qPCR of the indicated mRNAs from sorted organoid FAS+GL7+B220+ GCBs (n=3 biological replicates, i.e., organoids fabricated with 3 different spleens) and GC B cells sorted from immunized mice (n=3). Values are shown as mean fold change mRNA levels ± SD of biological triplicates normalized to the housekeeping gene *Hprt1* and further normalized to mean mRNA levels in NB cells. t test GCB vs. NB, *p<0.05, **p<0.01, ***p<0.001. (**F**) Splenocytes from immunized mice and organoids after 4 days in culture were permeabilized and stained for BCL6 using a fluorochrome-conjugated anti BCL6 antibody, and co-stained for GL7, FAS, IgD and B220 to identify GC B cells (FAS+GL7+B220+) and NB (FAS-GL7-IgD+B220+). The flow cytometry plot shows one representative sample of 3. The histogram shows the mean fluorescence intensity (MFI) ± SEM of BCL6 in NB, in vivo GCB and organoid GC B populations (n=3 per group) quantified by flow cytometry. (**G**) Principal component analysis of RNA-seq profiles of FAS+GL7+B220+ GC B cells sorted from immunized mice (*in vivo* GCB, n=6 mice); FAS+GL7+B220+ organoid GC B cells after 4 and 6 days in culture

(organoid GCB d4, n=4 biological replicates, and d6, n=3 biological replicates, i.e., organoids fabricated with 4 and 3 different spleens, respectively), and its respective 2D cultures (2D GCB d4, n=4 biological replicates, and d6, n=3 biological replicates). (**H**) Average ± SEM of percentage of proliferating organoid and 2D GC B populations (proliferation dye eFluor670+ GL7+FAS+B220+) of B cells in culture for the indicated days, quantified by flow cytometry as indicated in (B) (n=3 organoids and 2D culture per time-point). (**I**) Flow cytometry plot of a representative organoid and 2D GC B population after 5 days in culture stained with annexin V to identify apoptotic cells. (**J**) Average ± SEM of percentage of annexin V positive cells from organoid and 2D GC B populations (n=3 each) quantified by flow cytometry as shown in (I).

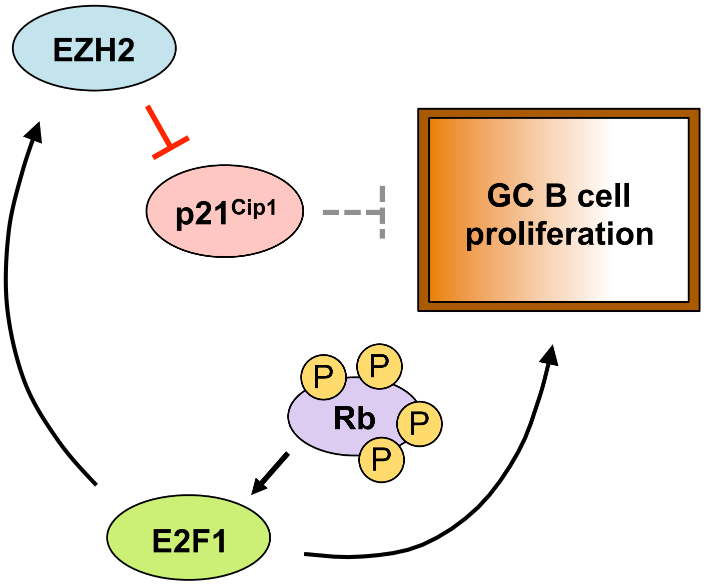

Supplementary Figure 5. Model of E2F1-EZH2-CDKN1A-Rb positive feedback loop

EZH2 controls GC B cell proliferation by suppressing CDKN1A, allowing cell cycle progression with a concomitant phosphorylation of Rb and release of E2F1. E2F1 positively regulates the expression of EZH2, resulting in a positive feedback loop.

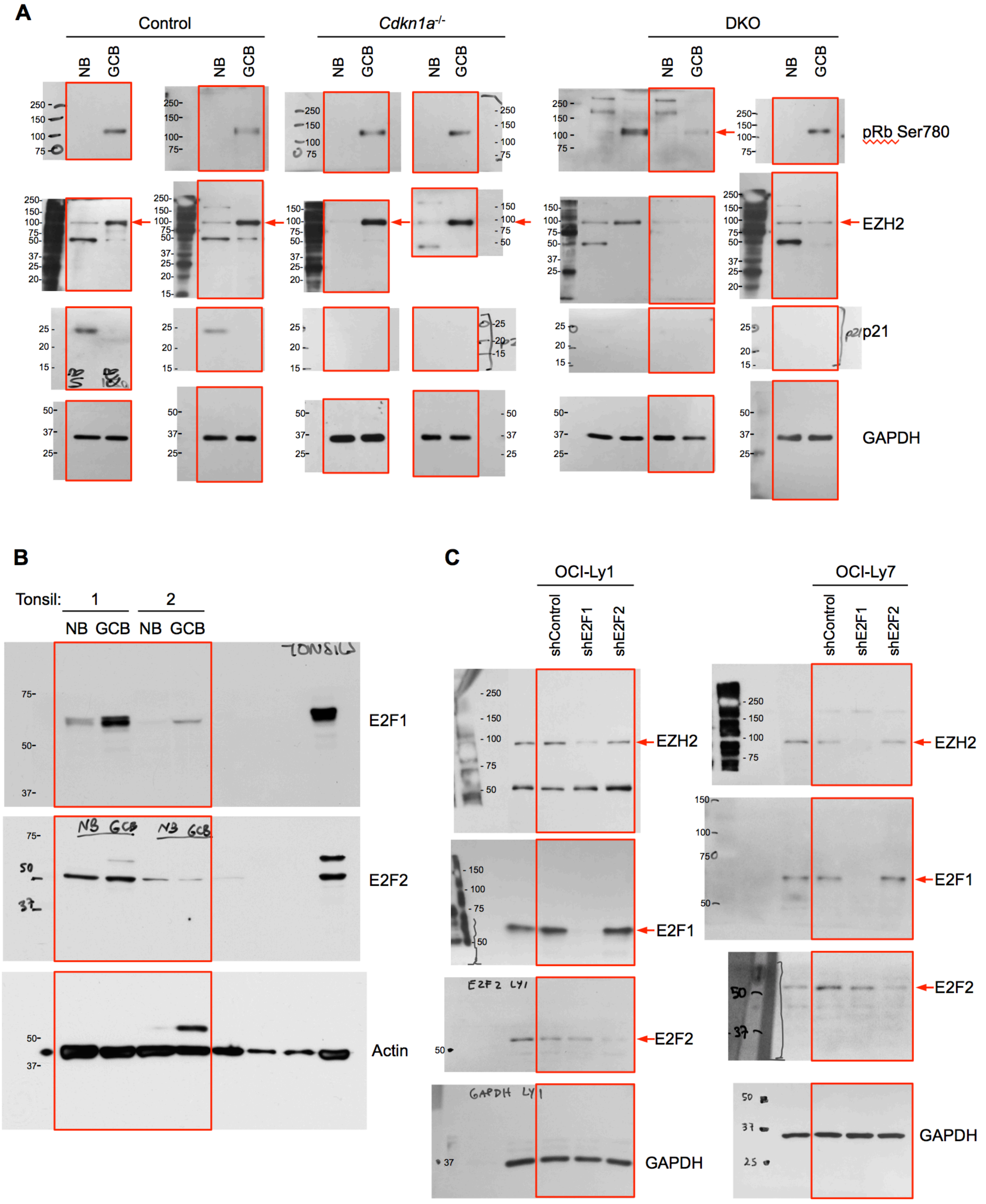

Supplementary Figure 6, related to figures 5 and 6.
Uncropped scans of western blots shown in main figures 5D (**A**), 6C (**B**), and 6D (**C**).

SUPPLEMENTARY TABLES

Supplementary Table 1. Primers used for qPCR

Used for	**Gene**		**Oligonucleotide (5'-3')**
cDNA	*Aicda*	Fwd	CCTCCTGCTCACTGGACTTC
		Rev	CAGGAGGTGAACCAGGTGAC
	Bcl6	Fwd	GCGAACCTTGATCTCCAGTC
		Rev	TGACTCTCACTGCTGCTTCG
	Cdkn1a	Fwd	GTGGCCTTGTCGCTGTCT
		Rev	TTTTCTCTTGCAGAAGACCAATC
	Ezh2	Fwd	ATCTGAGAAGGGACCGGTTT
		Rev	GCTGCTTCCACTCTTGGTTT
	E2f1	Fwd	AGAAACGGCGCATCTATGAC
		Rev	CTTCAAGCCGCTTACCAATC
	E2f2	Fwd	CCAAAAGGAAGTTGGACCTG
		Rev	CGACGTGTCATAGCGTGTCT
	Hprt1	Fwd	TATGCCGAGGATTTGGAAAA
		Rev	AATCCAGCAGGTCAGCAAAG
ChIP	*CDK1*	Fwd	TCTCCCGACTGGAGGAGAG
		Rev	ATCGGGTAGCCCGTAGACTT
	CDKN1A	Fwd	CAGTGGACCTCAATTTCCTCA
		Rev	AAAACGATGCACCTCTCTGC
	EZH2_1	Fwd	CGCCGTCTCTTTGTTCTTTC
		Rev	GTTCCCGCCACCTATCCT
	EZH2_2	Fwd	GGAAGCCAAGTTTGAACCAG
		Rev	CACAGCTGAGCCGACCTC
	Negative control region 1	Fwd	ATAACCCCAACGTGTCTTGC
		Rev	CCTCTGGCATCAGACTCTCC
	Negative control region 2	Fwd	AACCTGCAAAACATGGTTATTT
		Rev	AATTTGCCCAAACAGCAAGT

Supplementary Table 2. Primers used for immunoglobulin mutation analysis

Locus		Oligonucleotide (5'-3')
JH4	Fwd	GTCAAGGAACCTCAGTCACCGTCT
	Rev	CAGACCTCTCTAGACAGCAACTACC
Sμ	Fwd	AATGGATACCTCAGTGGTTTTTAATGGTGGGTTTA
	Rev	GCGGCCCGGCTCATTCCAGTTCATTACAG
VHJ558	Fwd	CGAGCTCTCCARCACAGCCTWCATGCARCTCARC
	Rev	TCTCAGCCGGCTCCCTCAGGG
Vλ1 - Jλ1,3	Fwd	GCCATTTCCCCAGGCTGTTGTGACTCAGG
	Rev	ACTCACCTAGGACAGTCAGCTTGGTTCC
