# Supplementary material for: EZH2 enables germinal centre formation through epigenetic silencing of CDKN1A and an Rb-E2F1 feedback loop
Source: Nat Commun. 2017 Oct 12;8:877. doi: 10.1038/s41467-017-01029-x (PMC5638898; doi:10.1038/s41467-017-01029-x)
Supplement: Supplementary file 2 — Peer review file [file 41467_2017_1029_MOESM2_ESM.pdf]

Editorial Note: This manuscript has been previously reviewed at another journal that is not operating a transparent peer review scheme. This document only contains reviewer comments and rebuttal letters for versions considered at Nature Communications.

REVIEWERS' COMMENTS:

Reviewer #1 (Remarks to the Author):

All concerns were addressed adequately in the response to reviewers, and as such is acceptable for publication.

Reviewer #3 (Remarks to the Author):

I was asked to comment on the criticism of referee 1 concerning the original version of the manuscript and the response of the authors to that criticism. The authors addressed all points raised by the referee adequately. Importantly, in response to major criticisms they performed substantial additional experiments. The results of these additional experiments nicely and convincingly further support the important story of the manuscript.

Reviewer #1 (Remarks to the Author):

All concerns were addressed adequately in the response to reviewers, and as such is acceptable for publication.

Reviewer #3 (Remarks to the Author):

I was asked to comment on the criticism of referee 1 concerning the original version of the manuscript and the response of the authors to that criticism. The authors addressed all points raised by the referee adequately. Importantly, in response to major criticisms they performed substantial additional experiments. The results of these additional experiments nicely and convincingly further support the important story of the manuscript.

Author Response: We thank the reviewers for their positive judgment of the revised version of our manuscript.
